# Ultrafast Laser-Induced Photothermoelectric Currents in Graphene Junctions in the Mid-Infrared

**DOI:** 10.1021/acs.nanolett.6c03809

**Published:** 2026-09-22

**Authors:** Nina Pettinger, Michel Panhans, Johannes Schmuck, Sebastian Loy, Xiaoyi Zhou, Chengye Dong, J. A. Robinson, Sergey Zherebtsov, Christoph Kastl, Frank Ortmann, Alexander W. Holleitner

**Affiliations:** † Walter Schottky Institute and Physics Department, 9184 Technical University of Munich, Am Coulombwall 4a, 85748 Garching, Germany; ‡ Munich Center for Quantum Science and Technology (MCQST), Schellingstr. 4, 80799 Munich, Germany; § TUM School of Natural Sciences, Department of Chemistry and Atomistic Modeling Center, Munich Data Science Institute, Technical University of Munich, 85748 Garching, Germany; ∥ Two-Dimensional Crystal Consortium, 8082 The Pennsylvania State University, University Park, Pennsylvania 16802, United States; ⊥ Department of Materials Science and Engineering, The Pennsylvania State University, University Park, Pennsylvania 16802, United States; # Department of Chemistry, The Pennsylvania State University, University Park, Pennsylvania 16802, United States; 7 Department of Physics, The Pennsylvania State University, University Park, Pennsylvania 16802, United States; 8 Department of Engineering Science and Mechanics, The Pennsylvania State University, University Park, Pennsylvania 16802, United States

**Keywords:** Photothermoelectric Currents, Graphene Junctions, Carrier Dynamics, Ultrafast Pump−Probe Photocurrent Spectroscopy, Dual Split Gate Device

## Abstract

We investigate the carrier dynamics in graphene junctions under mid-infrared excitation using ultrafast pump–probe photocurrent spectroscopy. We utilize dual split gate devices to demonstrate that the photothermoelectric effect can dominate the photocurrent response of graphene also for a mid-infrared femtosecond excitation. Graphene retains its broadband photocurrent response in this spectral region, but the photocurrent relaxation time increases from ∼2 ps below 8–9 μm up to 3 ps at longer mid-infrared wavelengths. The absence of a pronounced phonon bottleneck in the decay dynamics at room temperature suggests an efficient interplay of electron–electron and electron–phonon scattering even for photon energies below the optical phonon energy in graphene. The observed wavelength dependence of the photocurrent relaxation times is consistent with energy-dependent theoretical relaxation times as derived from a microscopic transport theory of graphene that includes electron–phonon coupling within a Holstein-Peierls Hamiltonian.

## Graphical abstract

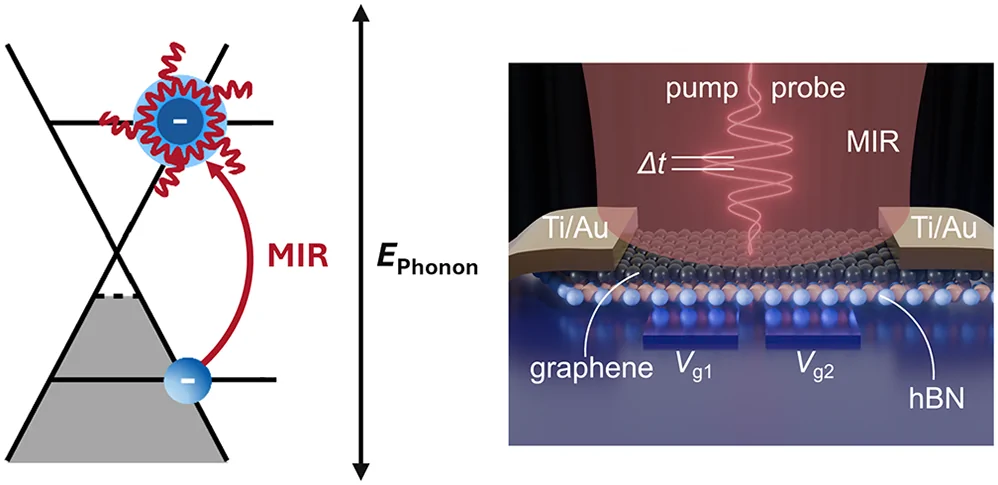

Graphene is an attractive platform for broadband optoelectronics because it uniformly absorbs light across an exceptionally broad spectral range.^1,2^ Therefore, unlike conventional semiconductors, graphene-based devices are not restricted to the visible or near-infrared: their operation naturally extends into the THz and mid-infrared (mid-IR), where applications in chemical sensing, free-space communications, and thermal imaging demand compact, ultrafast and sensitive detectors.^3,4^ To date, however, most demonstrations of ultrafast graphene photodetectors have focused on the near-infrared spectral range, reporting picosecond-scale response times.^5–15^ Extending such performance into the mid-IR spectral range remains largely unexplored.

At a fundamental level, understanding the relaxation processes of photoexcited carriers is essential for optimizing such graphene-based optoelectronic devices. The general picture of carrier excitation and relaxation^16–25^ in graphene can be summarized in the following way. Upon light absorption, electrons in graphene are driven out of equilibrium into a nonthermal distribution. Next, the excited electrons and holes thermalize into an ensemble of hot carriers, usually via fast electron–electron and Auger interactions (less than 100 fs).^23–26^ For slightly or undoped graphene, additionally, interband impact ionization processes play a role and can lead to a significant carrier multiplication in the conduction band.^23^ These hot carriers then dissipate their excess energy through interactions with the lattice, which is predominantly considered to be a defect-mediated coupling to acoustic phonon^27^ (or supercollision) cooling^28^ and a direct coupling to optical phonons.^25^ While the optical phonon emission process itself can generally be considered very efficient, with electron–phonon scattering times on the order of or below 100 fs, the overall relaxation and cooling rate appears to be intrinsically limited by a so-called hot phonon bottleneck, which slows down the equilibration of the hot optical phonons with the lattice via anharmonic decay into acoustic phonons, and thereby the overall electron cooling rate, to several ps.^21^ Moreover, the large optical phonon energies in graphene (on the order of 160–200 meV) open the possibility of a second considerable cooling bottleneck in undoped graphene, in particular for a mid-IR photoexcitation (a photon energy of 160 meV in vacuum corresponds to a wavelength of 7.7 μm): when the excess energy of the excited, thermalized electrons becomes comparable to the optical phonon energy, their cooling dynamics are expected to slow down significantly,^29^ with reported relaxation times on the order of 300 ps at low temperatures.^25^ While such a bottleneck is considered advantageous for a possible hot carrier extraction in solar cell devices, it would be an intrinsically limiting factor for ultrafast mid-IR to THz detectors. Lastly, in graphene encapsulated with hexagonal boron nitride (hBN), as is common for high-mobility and high-performance devices, additional cooling routes may emerge by interactions with the surrounding polar dielectric, such as coupling to surface polar phonons or hyperbolic phonon polaritons.^30–32^ These highly confined, long-lived infrared modes may efficiently accept energy from hot carriers in the adjacent graphene layer, thereby offering remote, substrate-mediated cooling pathways that compete with the more intrinsic optical phonon emission.^33,34^

Here, we investigate the ultrafast photocurrent generation in graphene junctions at room temperature and under a mid-IR excitation (5 μm – 12 μm). Using a combination of time-integrated and time-resolved photocurrent measurements, we find that the mid-IR photocurrent response of the graphene devices is governed by photothermoelectric currents, which maintains an ultrafast broadband photoresponse with photocurrent relaxation times on the order of a few picoseconds.

We fabricate graphene-based *p-n* junction devices on a silicon/silicon dioxide (Si/SiO_2_) substrate and probe the carrier dynamics by a mid-IR, time-resolved photocurrent autocorrelation scheme (Figure 1a, see Supporting Information). The junction is defined by a pair of platinum (Pt) split-gates. A hexagonal boron nitride (hBN) flake transferred onto the gates acts as a dielectric spacer. A monolayer graphene sheet with source-drain contacts serves as the active material. Figure 1b shows an optical image of the final device structure with the different layers highlighted in false color. For all photocurrent measurements, we apply a bias voltage *V*_sd_ at the source contact, and we measure the (photo)­current by a transimpedance amplifier at the drain contact as a function of gate voltages *V*_g1_, *V*_g2_ on the two split-gates. The two-terminal device resistance without any laser excitation as a function of the common gate voltage *V*_g_ = *V*_g1_ = *V*_g2_ (Figure 1c) and the individual gate voltages *V*_g1_ and *V*_g2_ (Figure 1d) shows a clear resistance peak. We interpret the resistance maximum to resemble the situation where the Fermi-energy is aligned to the Dirac point of the graphene. Notably, we find negligible hysteresis upon forward and reverse voltage sweeps demonstrating a high quality of the gate dielectric (Figure 1c).

**Figure 1:**
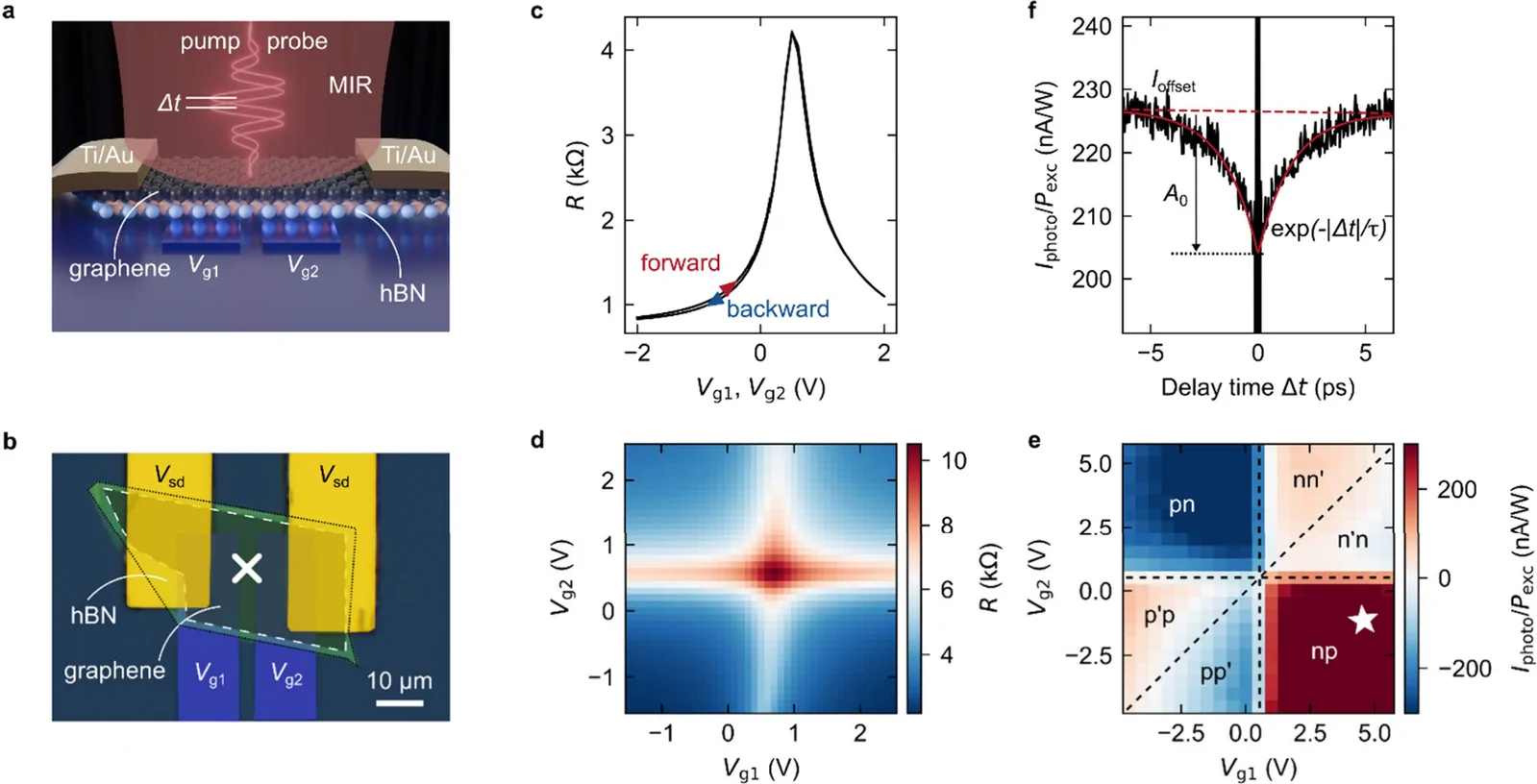
**Photothermoelectric photocurrent response of dual-gated graphene.** (a) Experimental scheme with a graphene-hBN heterostack contacted by two gold electrodes on top of two platinum split gates. The latter allow applying the gate voltages *V*_g1_ and *V*_g2_ to the heterostack. The graphene is excited by mid-infrared (mid-IR) pump and probe pulses with a time-delay *Δt* in-between. Bias voltage *V*_sd_ is zero for all shown pump–probe experiments. (b) Optical image of a graphene-hBN heterostack with split gates (highlighted in blue), ∼ 60 nm thin hBN (highlighted in green), monolayer graphene (highlighted in gray with white dashed outlines), and gold contacts (highlighted in yellow). White cross indicates the laser position. (c) Two-terminal resistance *R* of graphene when varying both gates simultaneously. (d) *R* vs *V*_g1_ and *V*_g2_. (e) Time-integrated photocurrent *I*_photo_ vs *V*_g1_ and *V*_g2_ for using just the pump laser at an excitation wavelength of λ_photon_ = 8 μm. The laser is positioned at the center of the device [cf. white cross in Figure 1(b)]. (f) Ultrafast photocurrent response *I*_photo_ vs *Δt* at λ = 8 μm, normalized to the excitation power *P*_exc_. In red: exponential fit to eq 1. The values of *V*_g1_ and *V*_g2_ are indicated with a white star in Figure 1(e).

For the photocurrent measurements, we focus a chopped, mid-IR laser beam onto the center of the device (white cross in Figure 1b). The resulting, time-integrated photocurrent is resolved with a standard lock-in detection (see Supporting Figure 1 for a photocurrent map). Figure 1e shows the gate-dependence of the photocurrent at *V*_sd_ = 0 V and at an excitation wavelength of λ_photon_ = 8 μm revealing a distinct multiple photocurrent sign change as a function of gate voltages, which is a characteristic signature of the photothermoelectric effect of hot-carrier-dominated transport in graphene *p-n* junctions (cf. Supporting Figure 2).^2,6,14,35,36^

To resolve the underlying carrier dynamics, we use a time-resolved, pump–probe photocurrent autocorrelation scheme.^5,7,9–13,15^ Figure 1f shows the photocurrent normalized to the excitation power as a function of the delay time *Δt* between the pump and probe pulses. The time-resolved photocurrent exhibits a dip around zero-time delay, which is consistent with the expected behavior of the photocurrent autocorrelation for the case of sublinear power scaling of the individual photocurrent pump- and probe-induced currents (cf. Supporting Figure 3). Near zero-time delay, we observe photocurrent oscillations due to the constructive and destructive interference of the copolarized pump and probe beams on the sample.^37^ We model the decaying photocurrent dynamics by an exponential function using

$$I_{\mathrm{photo}}=A_{0}\cdot \exp\left(-|\Delta t|/\tau \right)+I_{\mathrm{offset}}$$ 1

with *A*_0_ the amplitude at zero-time delay (*Δt* = 0) and τ the characteristic photocurrent relaxation time. The term *I*_offset_ accounts for the background current, which is present because we demodulate the photocurrent only at the chopping frequency of the pump beam (see Supporting Information). Additionally, we include a linear background in the fitting procedure that accounts for slow drifts during the measurement.

We note that we reproduced the ultrafast photothermoelectric currents on another graphene *p-n* junction device (Supporting Figure 4). Moreover, graphene devices on a single SiO_2_ back gate show the ultrafast mid-IR photocurrent response as well (Supporting Figure 5), where we interpret the dips again to correspond to the photothermoelectric effect of hot-carrier-dominated transport in graphene.^2,5,6^ Supporting Table 1 summarizes all seven samples studied within the scope of this work.

Figure 2 presents the wavelength- and gate voltage-dependent fitting parameters extracted from the ultrafast photocurrent measurements. For each configuration of split gate voltages (*V*_g1_, *V*_g2_), an ultrafast photocurrent measurement as a function of delay time (Figure 1f) is analyzed. The fit results, using the fit function in eq 1, are shown for the wavelengths 5 μm – 12 μm. Specifically, we map the amplitude *A*_0_ (Figure 2a), the photocurrent relaxation time τ (Figure 2b), and the offset current *I*_offset_ (Figure 2c). Note that if the photocurrent amplitude *A*_0_ does not exhibit a clear dip or peak, we cannot extract a meaningful value for τ. Such data points are set to be white in Figures 2a and 2b. The offset current *I*_offset_, which corresponds to the time-integrated photocurrent, reproduces the distinct multiple photocurrent sign change as a function of gate voltages as a characteristic of the photothermoelectric effect for the full range of the investigated excitation wavelengths (5 μm – 12 μm, Figure 2c). The time-resolved amplitude *A*_0_ (Figure 2a) follows a similar pattern but sign-reversed: if *I*_offset_ is positive, a dip in *I*_photo_(*Δt*) (cf. Figure 1f) will correspond to *A*_0_ being negative, and vice versa (cf. Supporting Figures 6 and 7), which agrees with the expected behavior for a power-saturated, sublinear photocurrent (cf. Supporting Figure 3). We find this observation holds well when the junction is biased into the *np* or *pn*-regime (cf. Figure 1e for the different doping regimes). Notably, when the junction is biased into the *nn′*, *n′n, p′p*, or *pp′*-regimes, we sometimes observe a peak-shaped rather than dip-shaped time-resolved photocurrent (cf. Supporting Figures 2, 8).^7^

**Figure 2:**
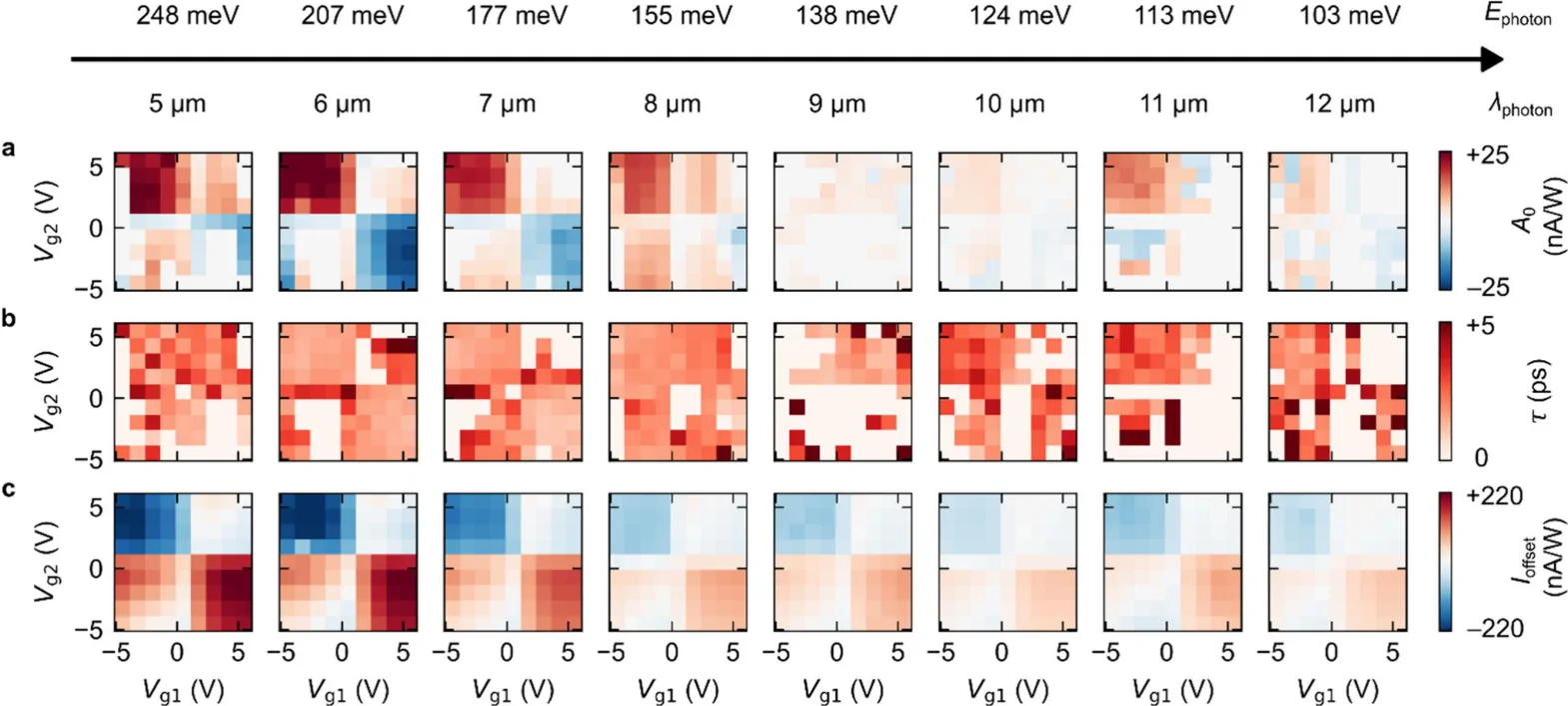
**Mid-IR wavelength dependence of ultrafast photothermoelectric photocurrent response.** Ultrafast photocurrent contributions are measured for wavelengths 5–12 μm as in Figure 1(f). (a) Fitted amplitude *A*_0_ vs *V*_g1_ and *V*_g2_. Values are normalized to the laser power per wavelength. (b) Fitted decay time τ vs *V*_g1_ and *V*_g2_. (c) Fitted offset current *I*_offset_ vs *V*_g1_ and *V*_g2_. Top panel shows the utilized mid-IR wavelength (5–12 μm) and corresponding photon energies for each of the columns below.

Our mid-IR pump–probe experiments allow us to connect the offset photocurrent *I*_offset_, which would be the quantity measured in a time-averaged measurement, to the ultrafast photocurrent dynamics via the time-resolved amplitude *A*_0_. Their close correspondence (cf. Figure 2) suggests that the photocurrent relaxation in our devices under mid-IR excitation is governed by the relaxation dynamics of hot carriers.^6^ Moreover, we observe that the photocurrent relaxation times are in the few ps regime, similar to the values as reported in literature for a near-IR excitation.^5,7–15^

Both *A*_0_ and *I*_offset_ decrease with increasing wavelength up to 10 μm (cf. Supporting Figure 10 for the second sample). To better highlight this behavior, Figure 3a depicts the mean amplitude *A*_0,mean_ with the values extracted from the top-left quadrant of Figure 2a, i.e. the *pn* regime, for each wavelength. Only nonzero entries, those measurements where a well-defined dip or peak is observed, are included in the average. The shaded error bands correspond to the error of *A*_0,mean_. Figure 3b summarizes the corresponding photocurrent relaxation time τ_mean_ averaged from the data in Figure 2b. The shaded areas denote again the estimate of the standard error for τ_mean_. Specifically, for wavelengths between 5 μm and 9 μm, the dominant optoelectronic relaxation time τ_mean_ remains nearly constant at ∼ 2 ps (Figure 3b and Supporting Figure 9), indicating a fast and wavelength-independent relaxation pathway of photoexcited charge carriers in this spectral range. Beyond 9 μm, however, the photocurrent relaxation time increases steadily, suggesting a slowdown of the dominant relaxation mechanism of the photoexcited charge carriers. We note that the wavelength dependence of the decay time τ_mean_ is consistent across the different measured device architectures: it is observed in both dual-gate devices (Figure 3b and Supporting Figure 9h) as well as in a single-gate device (Supporting Figure 9g).

**Figure 3:**
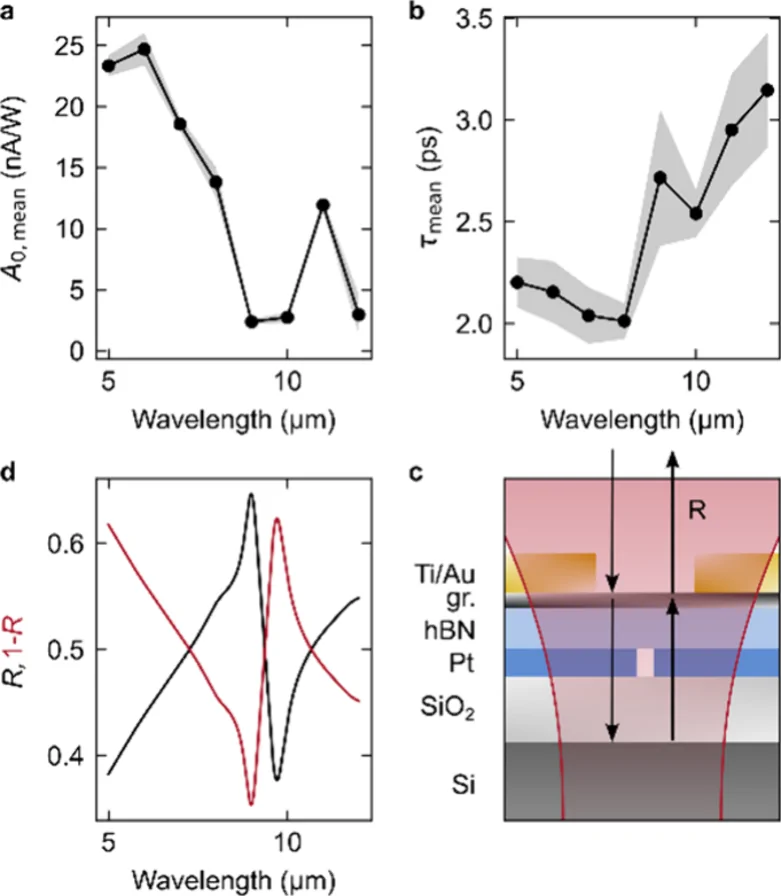
**Wavelength-dependent optoelectronic characteristics of the dual-gated graphene heterostack.** (a) Mean value of the fitted amplitude *A*_0_ vs photon wavelength. (b) Mean value of the fitted relaxation time τ vs photon wavelength. (c) Schematic side-view of the overall heterostack including the Si/SiO_2_-substrate, which allows calculation of the reflection *R* of the incident light. (d) Calculated reflection *R* (black) and the term 1 – *R* (red) of the overall heterostack as shown in Figure 3(c) vs photon wavelength. See text for details.

To understand the wavelength dependence of the photocurrent amplitude, we estimate the dielectric properties of the full heterostructure stack (Figure 3c) using the transfer matrix method (Supporting Note 1).^38,39^ The calculated reflection *R* (Figure 3d) increases monotonically up to approximately 9 μm, which explains the overall trend that both *A*_0_ and *I*_offset_ decrease in this wavelength range (Supporting Figure 10). We note, however, that this simple calculation neglects lateral finite-size and possible antenna effects.

To rationalize the measured photon-energy dependence of the decay times, we turn to a microscopic transport description of charge carriers in graphene that explicitly accounts for electron–phonon coupling and that allows us to extract energy-dependent relaxation times within linear-response theory. We describe charge transport over a range of energies accessed by mid-IR excitation through explicit propagation of quantum states in the coupled electron–phonon Hilbert space, while accounting for electron–phonon interactions with optical phonon modes (i.e., propagation of electron–phonon wave packets). This provides access to carrier relaxation processes and possible phonon-dressing effects upon excitation into the conduction band (Figure 4a).

**Figure 4:**
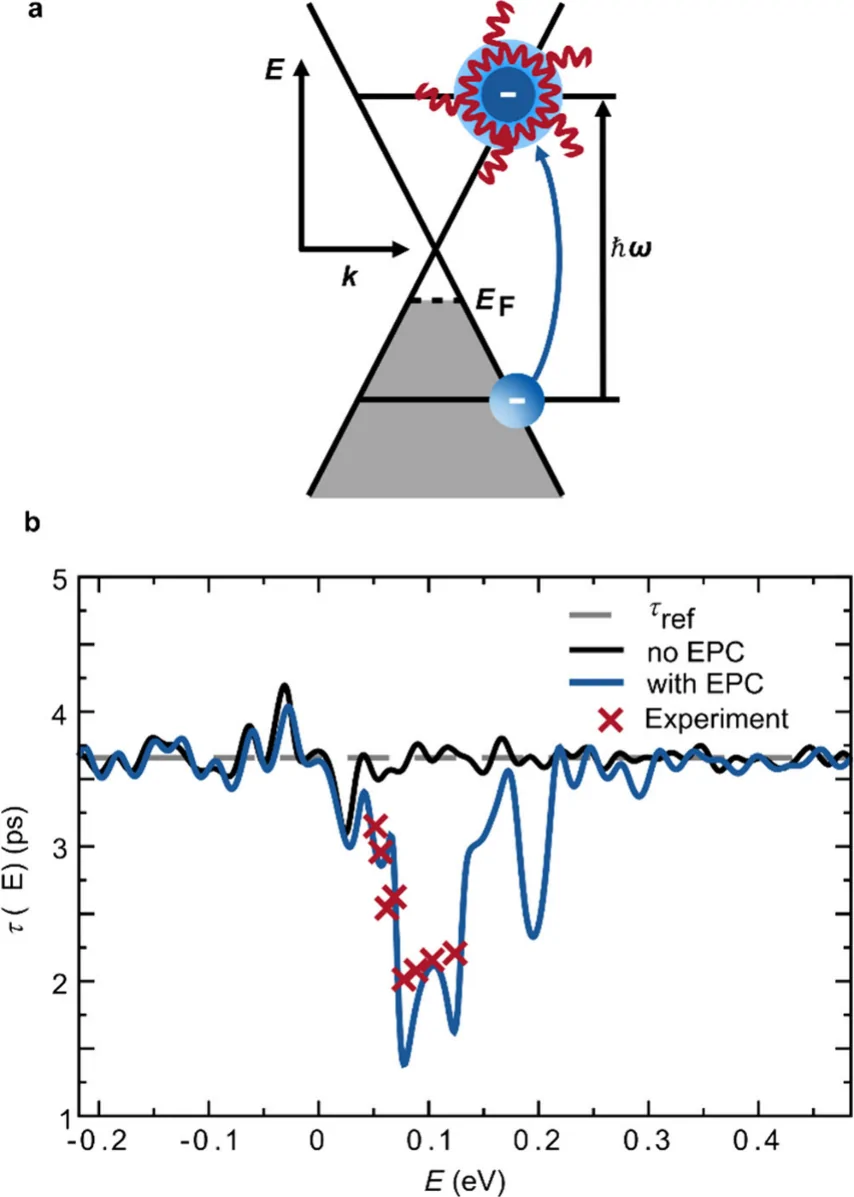
**Relaxation, hot carrier, and polaron effects in graphene.** (a) Schematic illustration of an excited electron that is coherently dressed with optical phonons leading to polaron dressing at time scales above several tens of fs. (b) Energy dependence of the experimental decay time (red crosses) compared to theoretical carrier relaxation times according to eq 2, including the case of absence of the electron–phonon coupling (no EPC, black) and the interacting case (with EPC, blue), where the results of LO and TO optical phonons are averaged. The energy axis is half the photon energy used in the experiment as sketched in (a). The dashed gray line represents the total simulation time for inelastic processes serving as a reference time scale. Note that all curves are aligned with their Dirac point at *E* = 0.

Interband transitions across the Dirac point create a nonequilibrium distribution of charge carriers with electrons at energies *E* = 1/2 · ℏω approximately above the CNP (Figure 4a). To determine the characteristic relaxation time at these energies, we evaluate the energy-dependent transport scattering time from the dc-conductivity in the zero-temperature limit. The underlying microscopic description of graphene is based on a Holstein–Peierls Hamiltonian that explicitly includes the coupling of charge carriers to optical phonon modes. The corresponding relaxation time as a function of energy^40^ is obtained as

$$\tau \left(E\right)=2\frac{\underset{T\to 0}{\lim}\sigma_{\alpha \alpha }^{\mathrm{dc}}\left(E,T\right)}{e^{2}D\left(E\right)v_{F}^{2}}$$ 2

where σ_*αα*_^dc^ is the diagonal component of the dc-conductivity tensor, *D*(*E*) the density of states of the Hamiltonian, and *v*_F_ the bare Fermi velocity of graphene at the Dirac point. Within this approach, the dc-conductivity is obtained from the time dependence of the electronic mean-square displacement Δ*x*_*a*_^2^(*t*), which is obtained from the real-time evolution of quantum states in the electron–phonon Hilbert space. The propagation is performed using a highly efficient numerical charge-transport method that fully accounts for electron–phonon interaction with optical phonon modes^41–43^ (see Supporting Notes 2 and 3 for details).

As shown in Figure 4b, this approach yields a pronounced energy dependence of the carrier relaxation time [eq 2]. For reference, the dashed gray line indicates the parametric inelastic time scale τ_ref_ at which the transport dynamics are investigated. In the absence of electron–phonon coupling (EPC), electrons propagate ballistically as reflected by a quadratic time dependence of the mean-square displacement Δ*x*_*a*_^2^(*t*) ∝ *t* ^2^ approximately equal for all energies *E* (black line). When the EPC is included, the relaxation time exhibits a marked reduction of the conductivity and hence *τ­(E)* at energies around *E* ≈ 0.1 eV, followed by a gradual recovery toward the ballistic limit at higher energies of ∼ 0.3 eV (blue line). Due to this interaction, the propagated electronic states naturally develop phonon dressing during the dynamics. For the parameters used in our simulation, this polaronic dressing develops on ultrafast time scales (∼ 20 fs), which corresponds to the characteristic time scale set by the optical phonon energies and is accompanied by a substantial renormalization of the carrier velocity of about 70%. We note that this renormalization value is consistent with previous angle-resolved photoemission spectroscopy studies on velocity renormalization in graphene^44,45^ and it is significantly larger than predictions using electron self-energy renormalization schemes leading to a velocity renormalization of 4% to 8%.^46^ The (dimensionless) EPC constant used in this work is 0.159 (see Supporting Note 2). Previous TR-ARPES studies report an about 15% larger value of 0.187.^45^ Note that the experimental reference reports the squared EPC constant (0.035). Therefore, the present microscopic theory captures independent experimental observations in several key aspects and therefore provides a reliable basis for explaining the mid-IR photocurrent measurements. Importantly, the electron–phonon interaction that governs the transport relaxation time also affects the temporal evolution of the nonequilibrium carrier distribution generated under optical excitation. In this sense, the renormalization effects captured in the transport simulations are expected to directly influence the photocurrent relaxation dynamics. This provides a consistent microscopic link between the calculated energy-dependent relaxation times and the experimentally observed decay times.

We first discuss the extent to which the microscopic transport simulations capture the experimentally observed photocurrent dynamics. As shown in Figure 4b, the calculated energy dependence of the theoretical carrier relaxation time covers the overall trend of the experimental photocurrent data very well (red crosses), including the pronounced minimum at intermediate energies. Assuming τ_ref_ to be 3.7 ps, the calculation yields relaxation times that are comparable in magnitude to the experimentally observed values of the photocurrent relaxation time and correctly capture their photon-energy dependence. This agreement suggests that, also under mid-infrared excitation, the interaction of hot charge carriers with optical phonons plays a central role in governing the photocurrent relaxation dynamics in graphene. Generally, the assumed value of τ_ref_ (3.7 ps) is on the same order as reported for purely optical mid-IR pump–probe experiments at similar photon energies below the optical-phonon energy at room temperature.^25^ The strong carrier–carrier interaction in graphene provides a hot carrier distribution on sub-ps to ps time scales, allowing hot electrons still to scatter efficiently with optical phonons.^25^ However, a one-to-one comparison of time scales as deduced from purely optical and photocurrent experiments is beyond the current work.

We further note that the presented ultrafast mid-IR photocurrent experiments with an electric detection are in between optical pump/probe experiments^22–26^ and lateral electrical transport experiments without an optical illumination conducted at the Fermi energy, where interactions with defects, charge impurities, and inhomogeneities of the sample can be dominating.^47–49^ Importantly, our samples show a considerable room temperature mobility of ∼ 25000 cm^2^V^–1^s^–1^, such that we can assume to be in a regime where the relaxation is not due to defect mediated processes such as supercollisions, but rather due the intrinsic optical phonon interactions and dynamics.^16^

We finally point out that for a photon energy below twice the value of *E*_F_, it is expected that the carrier distribution can be heated via free-carrier absorption processes and experimentally, that the relaxation time further increases.^25^ For this purpose, we investigated the dual-gated devices also further away from the charge neutrality point (*V*_g1_ and *V*_g2_ = ± 10 V cf. Supporting Figure 11), corresponding to a Fermi energy of 0.3 eV, which is above the investigated photon energies (Figure 2). However, we observe that the ultrafast mid-IR photocurrent response vanishes entirely. This behavior is consistent with our comparison sample of epitaxial graphene on SiC, which has a Fermi energy of 0.4 eV and likewise shows no ultrafast mid-IR signal within the given signal-to-noise ratio (cf. Supporting Figure 12). Future experiments might be able to resolve such photocurrent dynamics at even lower photon energies and/or a higher Fermi-energy and/or in other materials.^50–52^

Generally, our results indicate that graphene is a promising platform for ultrafast photodetection in the mid-infrared spectral range. The measured photothermoelectric currents show that mid-infrared illumination can be converted into an electrical signal on a few-picosecond time scale. Taking Johnson noise as the noise-floor limit, the ultrafast amplitude as in Figure 1f implies a detectivity limit on the order of ∼10^3^ Hz^1/2^/W. Relative to established mid-IR detectors, this value is low: type-II superlattice photodetectors report 10^13^ Hz^1/2^/W, HgCdTe detectors 10^10^ Hz^1/2^/W,^11,23^ and quantum dot detectors 10^8^–10^10^ Hz^1/2^/W.^53,54^ Nevertheless, detectivity could be improved by increasing device absorbance using metasurface structures on top of the graphene, suggesting detectivities up to 10^11^ Hz^1/2^/W.^55,56^ In addition, graphene offers practical advantages, including a straightforward fabrication and stability against gases; for example, all measurements shown were performed under dry N_2_-gas.

To summarize, we explore the ultrafast photocurrent dynamics in graphene junctions under mid-infrared excitation by employing a femtosecond pump–probe photocurrent spectroscopy. Using dual split-gate devices, we show that the photothermoelectric effect can govern the ultrafast photocurrent response even for mid-infrared excitation, underscoring the robustness of thermoelectric photocurrent generation beyond the commonly studied near-infrared and visible regimes. Notably, we do not observe signatures of a pronounced phonon bottleneck at room temperature. This indicates efficient energy relaxation and transport dynamics mediated by the combined action of electron–electron and electron–phonon scattering, even when the excitation energy is below the optical phonon energy in graphene. The measured trend in relaxation times with wavelength is consistent with energy-dependent relaxation times predicted by a microscopic transport theory incorporating the electron–phonon coupling within a Holstein-Peierls framework.

## Supplementary Material



## Data Availability

The data as discussed in the current study is available from the authors upon reasonable request.

## Notes

The Supporting Information includes further experimental details, the theoretical model Hamiltonian as well as the charge transport approach, and further data as measured on all investigated samples. (PDF)
