# Supplementary material for: Ultrafast Laser-Induced Photothermoelectric Currents in Graphene Junctions in the Mid-Infrared
Source: Nano Lett. 2026 Sep 22;26(39):13627–34. doi: 10.1021/acs.nanolett.6c03809 (PMC13651623; doi:10.1021/acs.nanolett.6c03809)
Supplement: Supplementary file 1 [file nl6c03809_si_001.pdf]

Supporting Information

Ultrafast photo-thermoelectric currents in graphene junctions in the mid-infrared

Nina Pettinger[1,2], Michel Panhans[3], Johannes Schmuck[1,2], Sebastian Loy[1,2], Xiaoyi Zhou[1,2], Chengye Dong,[4,5] J.A. Robinson[4,5,6,7,8], Sergey Zherebtsov[1,2], Christoph Kastl[1,2], Frank Ortmann[3*], Alexander W. Holleitner[1,2*]

[1] Walter Schottky Institute and Physics Department, Technical University of Munich, Am Coulombwall 4a, 85748 Garching, Germany

[2] Munich Center for Quantum Science and Technology (MCQST), Schellingstr. 4, 80799 Munich, Germany

[3] TUM School of Natural Sciences, Department of Chemistry and Atomistic Modeling Center, Munich Data Science Institute, Technical University of Munich, 85748 Garching, Germany.

[4] Two-Dimensional Crystal Consortium, The Pennsylvania State University, University Park, PA, USA

[5] Department of Materials Science and Engineering, The Pennsylvania State University, University Park, PA, USA

[6] Department of Chemistry, The Pennsylvania State University, University Park, PA, USA

[7] Department of Physics, The Pennsylvania State University, University Park, PA, USA

[8] Department of Engineering Science and Mechanics, The Pennsylvania State University, University Park, PA, USA

[*] Contact Email: holleitner@wsi.tum.de, Frank.Ortmann@tum.de

The PDF file includes:

Supporting Note 1-3

Supporting Figs. 1 to 13

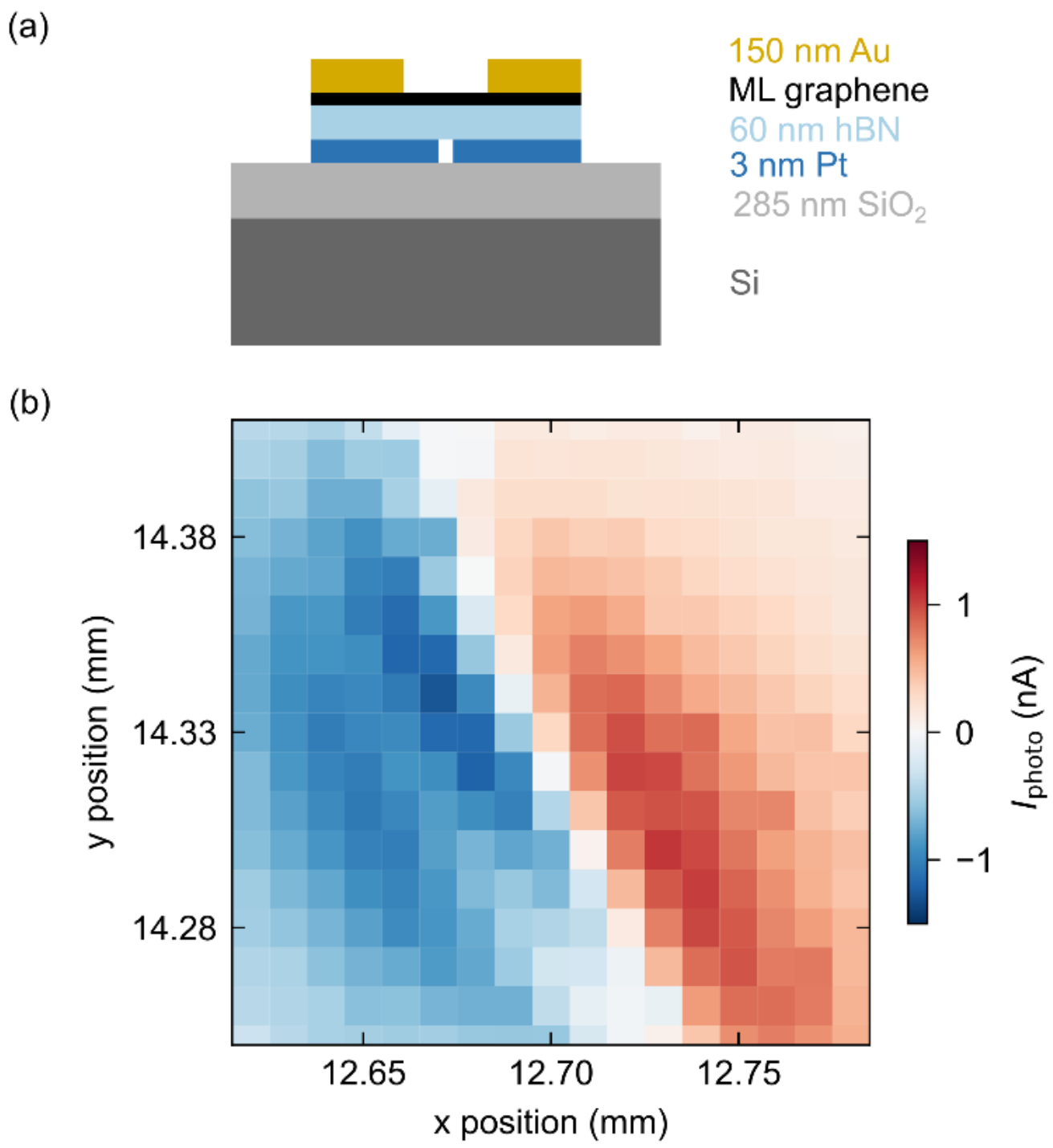

Supporting Figure 1. Split gate sample Graphene 1. (a) Schematic side-view of the sample geometry. (b) Spatially resolved photocurrent (PC) map (λ = 8 µm and V_{g1} = V_{g2} = V_{sd} = 0 V). Each pixel length equals 10 µm.

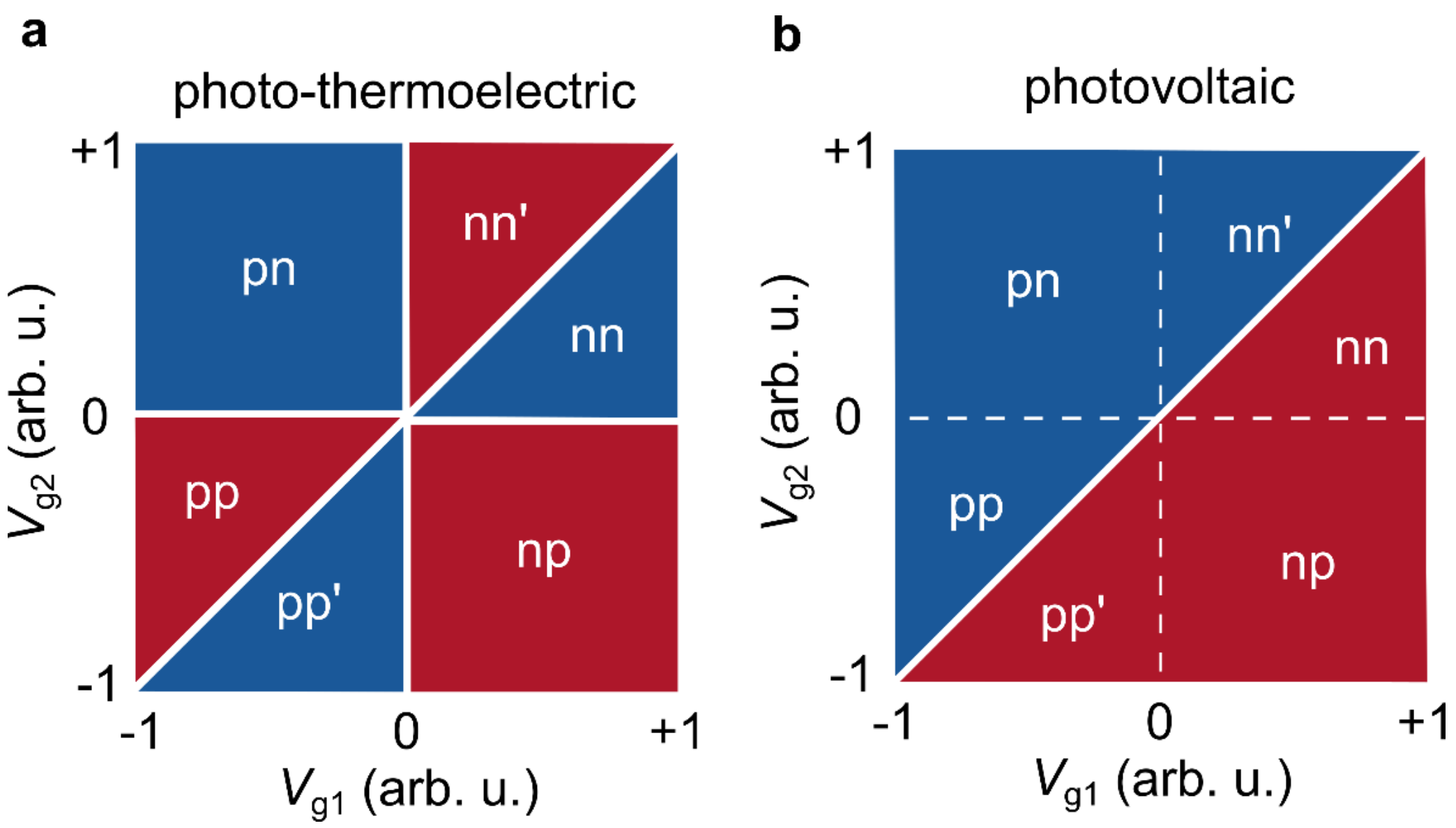

Supporting Figure 2. Scheme of the photo-thermoelectric and photovoltaic responses in a dual-gated graphene device as a function of the gate voltages V_{g1} and V_{g2}. The gate voltages are applied to dual split gates as sketched in Fig. 1a of the main manuscript. (a) Photo-thermoelectric response with a multiple photocurrent sign-change of the photocurrent (red – positive and blue – negative current) as introduced in ref.[[1]]. (b) Photovoltaic response, where only two sections with either positive or negative currents evolve.

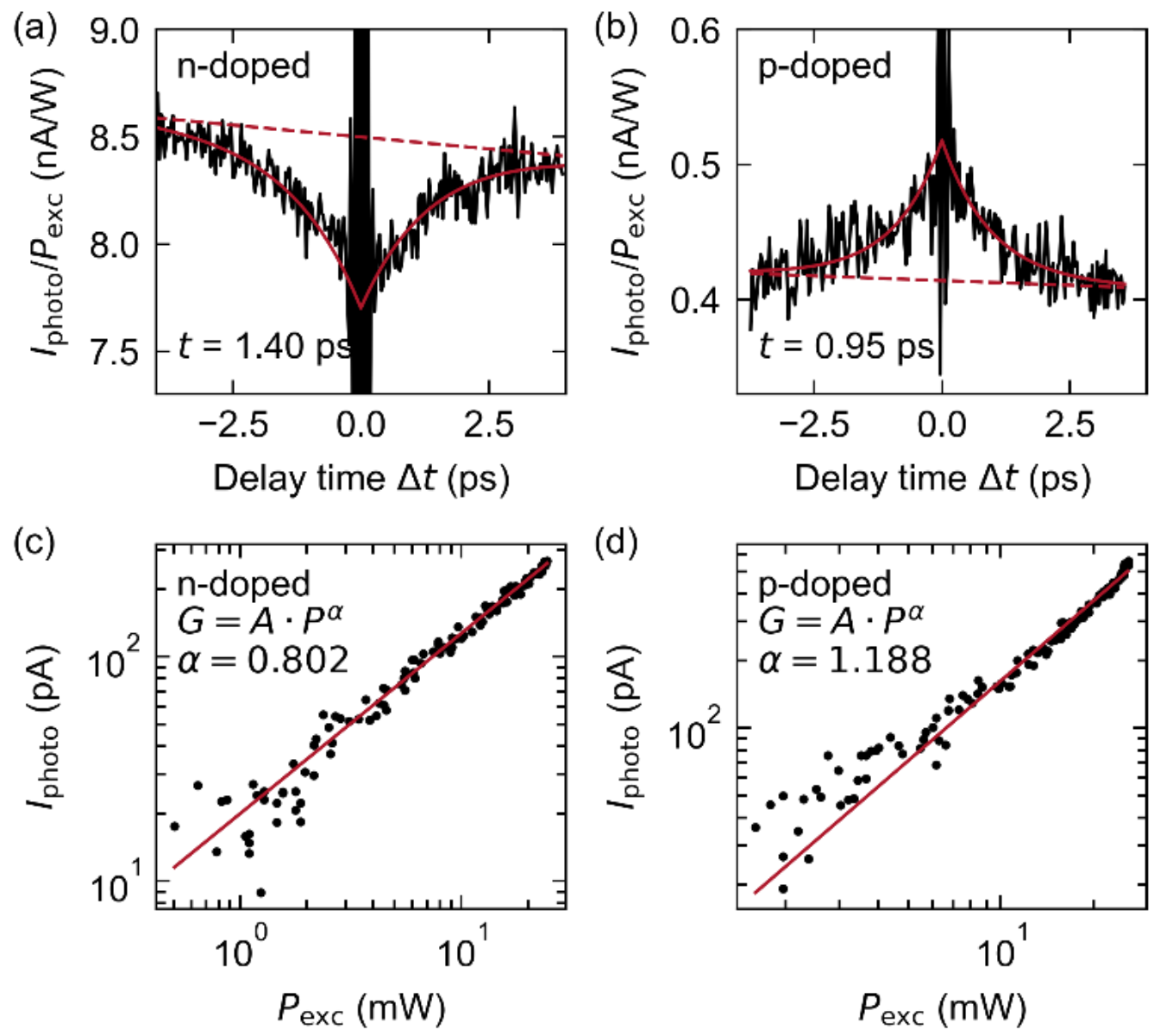

Supporting Figure 3. Power dependence of the photocurrent and corresponding photocurrent autocorrelation. (a), (b) Exemplary ultrafast photocurrent measurements for (a) *n*-doping and (b) *p*-doping. (c), (d) Double-logarithmic plot of the photocurrent as a function of excitation power for (c) *n*-doping and (d) *p*-doping. We observe that for *n*-doping (*p*-doping) the signal shows a dip (peak) and a sublinear (superlinear) power dependence with an exponent of 0.802 (1.188) in accordance with literature[2,3].

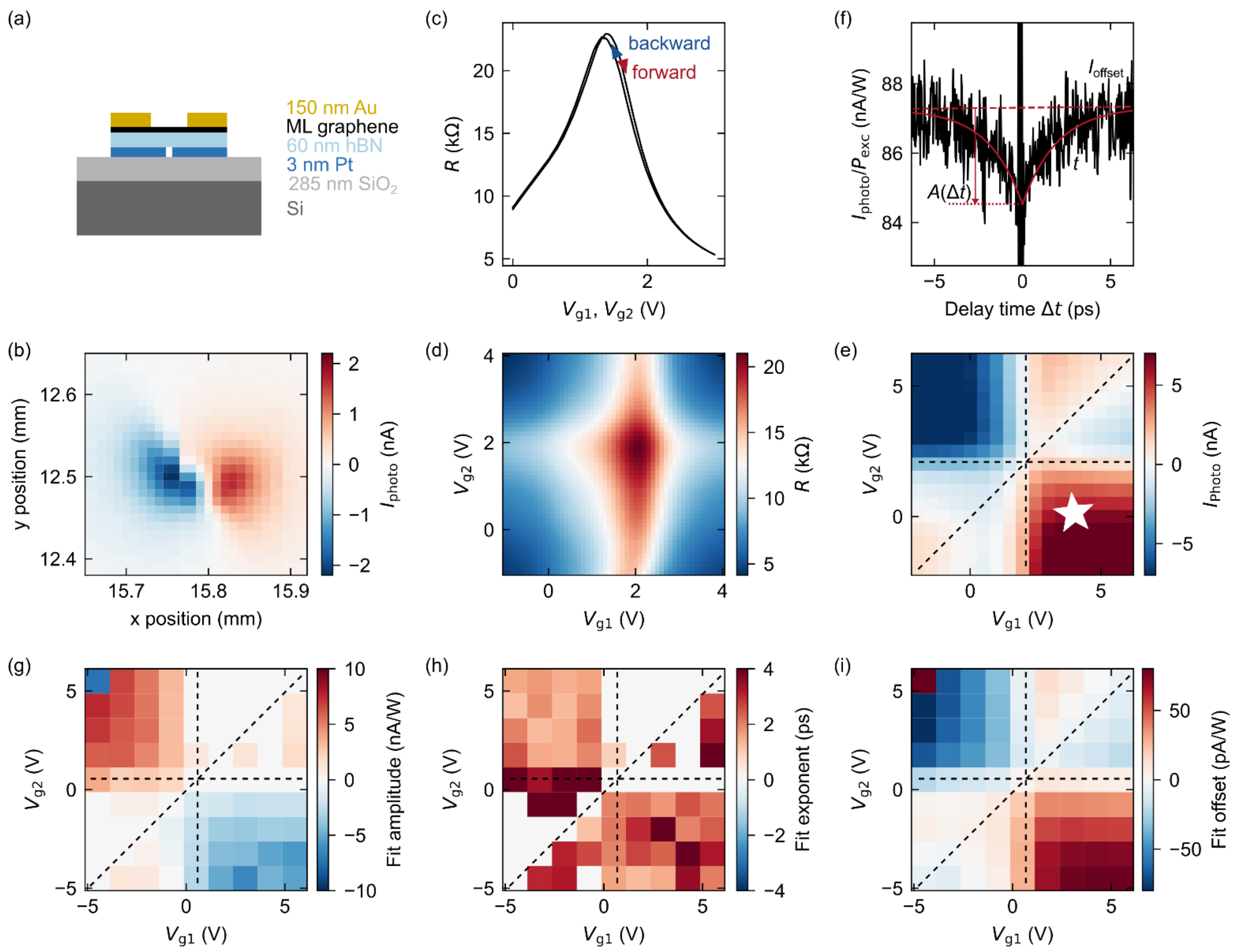

Supporting Figure 4. Sample 2 (split gate sample with graphene). (a) Schematic side-view of the sample geometry. Identical to the sample geometry of sample Graphene 1 (main part). (b) Spatially resolved photocurrent (PC) map. Each pixel length equals 10 µm. Taken with λ = 8 µm and V_{g1} = V_{g2} = V_{sd} = 0 V. (c) Resistance R of graphene when varying both split gate voltages simultaneously. (d) Resistance R map of graphene when varying both split gates individually. (e) Photocurrent I_{Pphoto} map when varying both split gate voltages individually, with λ = 8 µm and V_{sd} = 0 V (f) Ultrafast photo response I_{photo} as a function of delay time Δt between pump and probe pulse, normalized with the excitation power P_{exc}, in black. Exponential fit $A(0)\ exp(-|\Delta t|\ /\ t) + I_{offset}$ in red. (g)-(i) Fit parameter maps. At each split gates point (V_{g1}, V_{g2}), an ultrafast photocurrent measurement as a function of delay time is taken. The fit results are shown: (g) amplitude $A(\Delta t = 0)$, (h) exponent t, (i) offset I_{offset}.

	Fabrication	Gate	Substrate	n_{2D}	Ultrafast MIR response	t (ps)	hBN thickness
Graphene 1	mech. exfol.	split gates	Si/SiO$_2$ (285 nm)	dep. on V_{g1}, V_{g2}	yes	1.55 +/- 0.21	60 nm
Graphene 2	mech. exfol.	split gates	Si/SiO$_2$ (285 nm)	dep. on V_{g1}, V_{g2}	yes	1.94 +/- 0.73	60 nm
Graphene 3	mech. exfol.	single gate	Si/SiO$_2$ (285 nm)	dep. on V_g	yes	2.65 +/- 0.09	-
Graphene 4	mech. exfol.	single gate	Si/SiO$_2$ (285 nm)	dep. on V_g	yes	2.82 +/- 0.58	-
Graphene 5	mech. exfol.	single gate	Si/SiO$_2$ (285 nm)	dep. on V_g	yes	2.40 +/- 1.79	-
Graphene 6	mech. exfol.	single gate	Si/SiO$_2$ (285 nm)	dep. on V_g	yes	1.03 +/- 1.04	-
Graphene 7	CVD grown	none	SiC	10×10^{12} cm^{-2}	no	-	-

Supporting Table 1. Overview of samples as shown in Supporting Figure 2. Graphene 1 corresponds to the sample shown in the main manuscript.

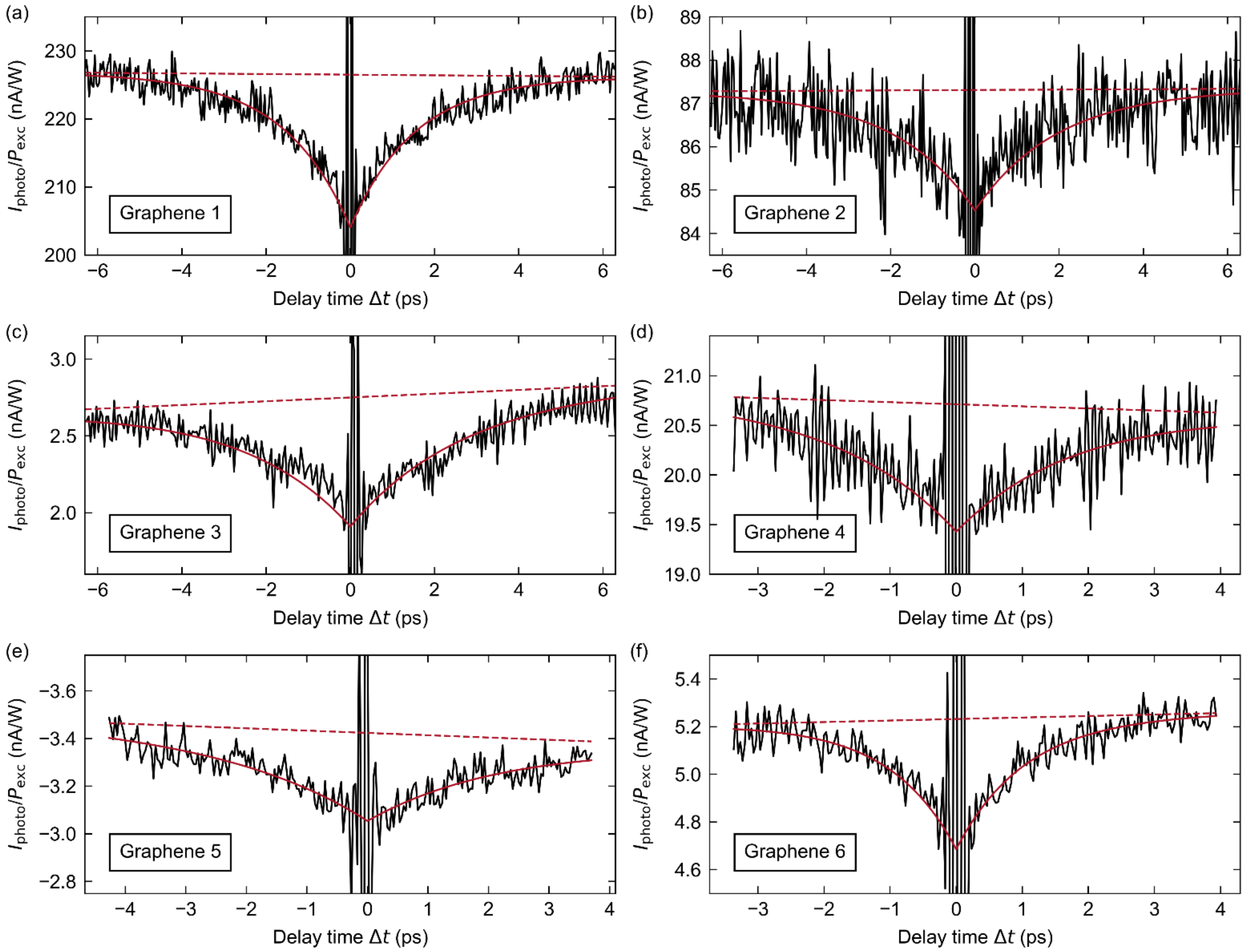

Supporting Figure 5. Overview of samples as defined in Supporting Table 1. Ultrafast photo response in (a) sample Graphene 1 (referred to as graphene sample in the main manuscript), (b) sample Graphene 2, (c) sample Graphene 3, (d) sample Graphene 4, (e) sample Graphene 5, (f) sample Graphene 6. Corresponding exponential fits (solid line) and linear backgrounds (dashed line) are shown in red.

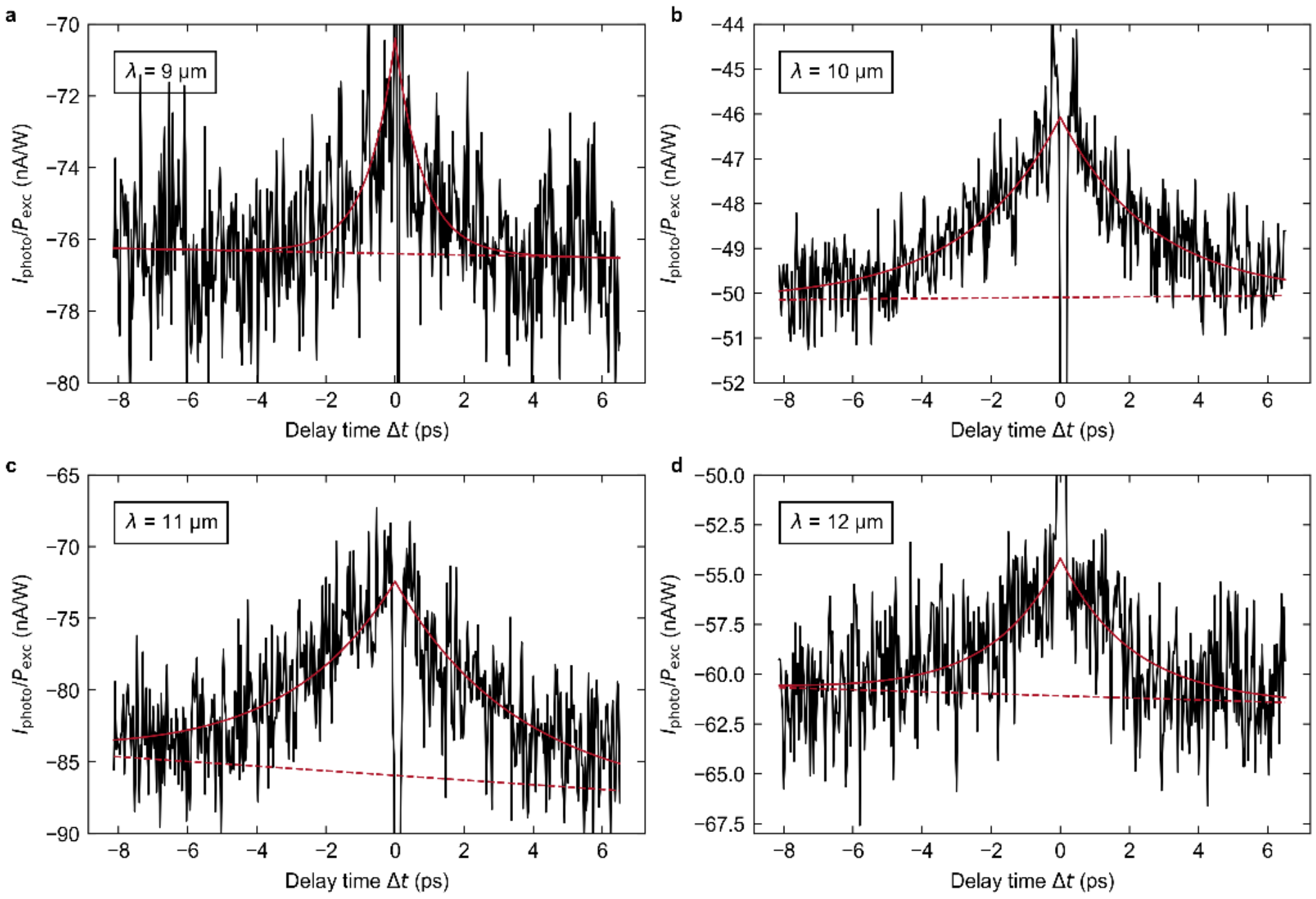

Supporting Figure 6. Original time-resolved photocurrent data of sample "Graphene 1" (cf. Supporting Table 1) measured in the doping regime pn regime (cf. Supporting Figure 2) for photon wavelengths 9 μm, 10 μm, 11 μm, and 12 μm. The red lines are fits to the data as discussed in the main manuscript.

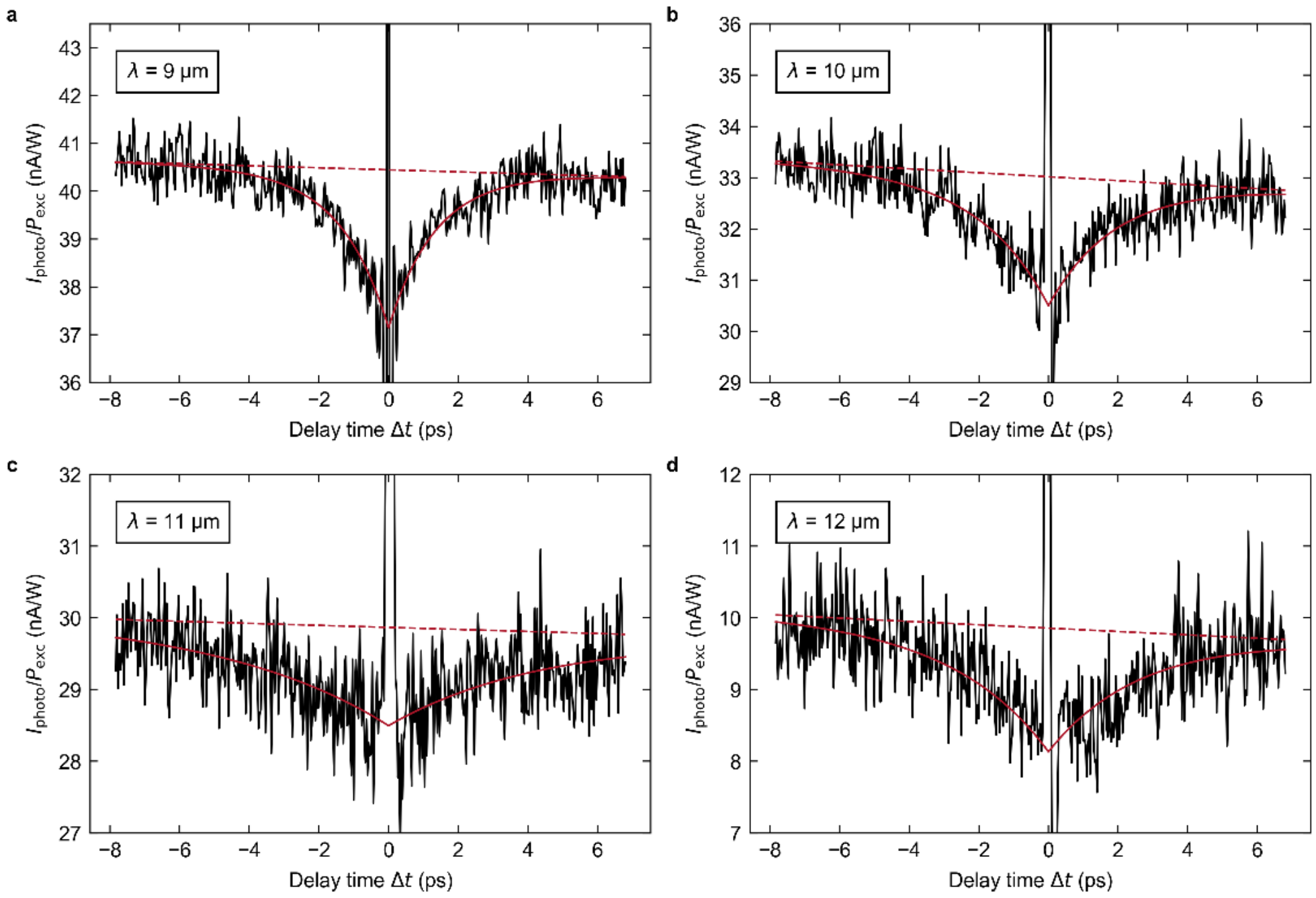

Supporting Figure 7. Original time-resolved photocurrent data of sample "Graphene 2" (cf. Supporting Table 1) measured in the doping regime np regime (cf. Supporting Figure S2) for photon wavelengths 9 μm, 10 μm, 11 μm, and 12 μm. The red lines are fits to the data as discussed in the main manuscript.

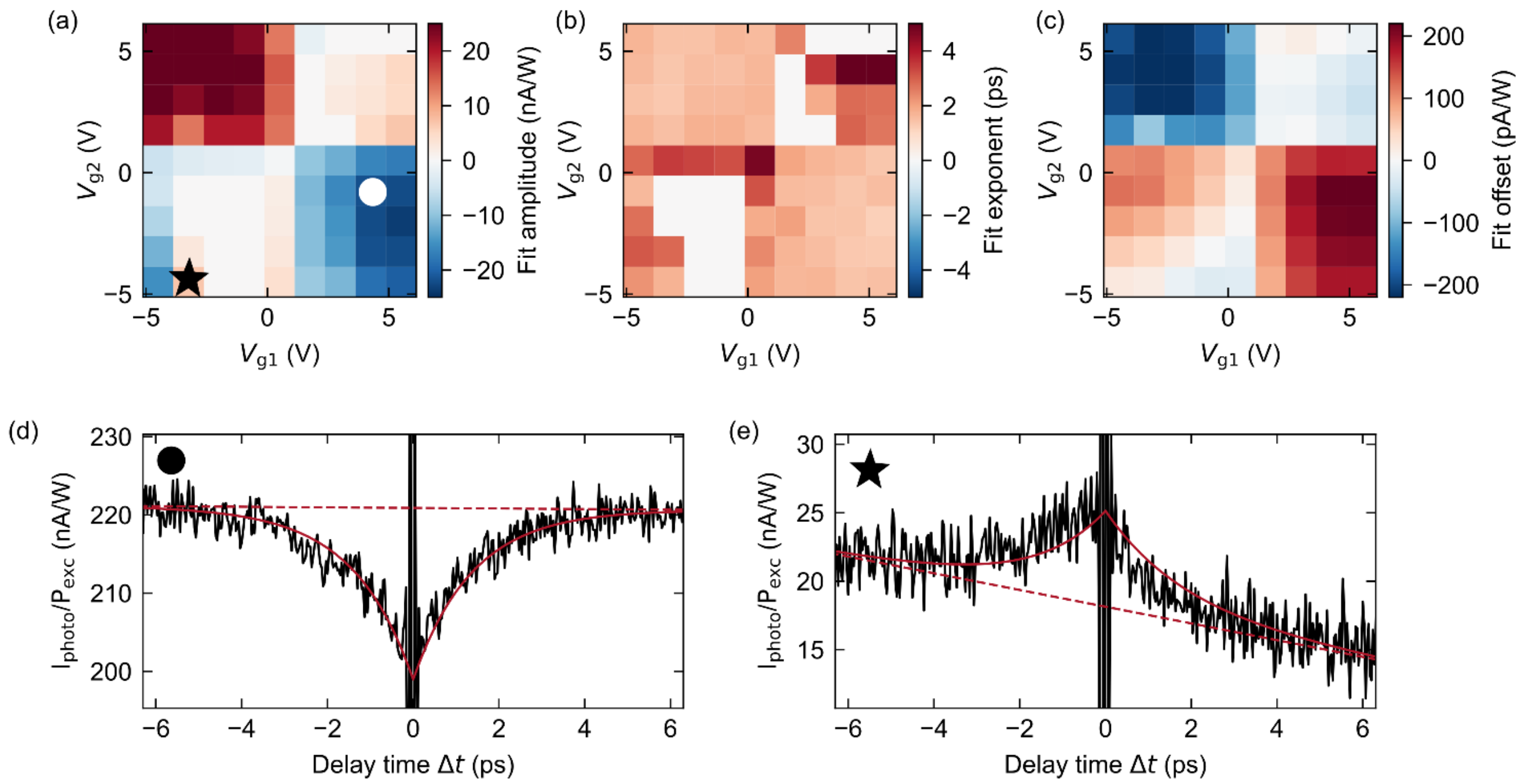

Supporting Figure 8. Dip- and peak-shaped photocurrent with time delay for λ = 6 µm. (a) Amplitude, (b) time constant, (c) offset as shown in Fig. 2. The circle and the star indicate the gate voltages (V_{g1} and V_{g2}) where the ultrafast photocurrent measurements (d) and (e) are recorded.

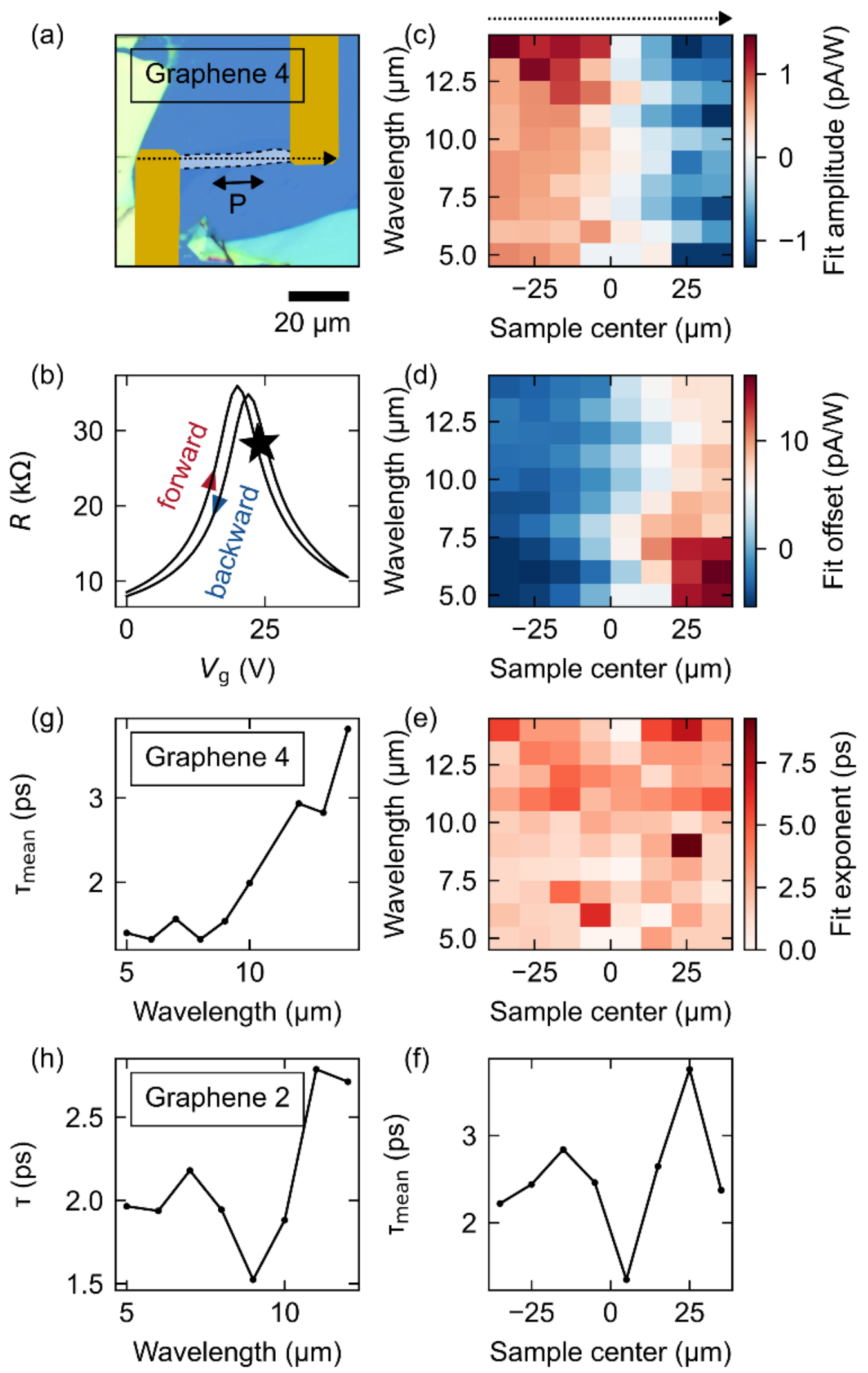

Supporting Figure 9. Spatial dependence for sample Graphene 4 (single gate graphene device). (a) Microscope image of the graphene device. The laser polarization *P* and the scan direction (dashed arrow) are indicated. (b) Transfer curve. The gate voltage used for the following measurements is indicated with a star. (c), (d), (e) Scan across the sample for different wavelengths. At each point, an ultrafast photocurrent measurement is taken and the exponential fits according to equation (1) of the main manuscript shown as maps: (c) amplitude $A(\Delta t = 0)$, (d) offset I_{offset}, (e) exponent τ. (f) Projection of the fit exponent in (e) onto the scan position axis. (g) Projection of the fit exponent in (e) onto the wavelength axis. (h) Wavelength dependence of the time constant for sample Graphene 2 (split gate), as a comparison. We note that also in single-gated graphene-based devices, the photo-thermoelectric and photo-voltaic photocurrents equally exist; particularly at interfaces e.g. at the graphene/metal interface close to the electrodes. Such interfaces can produce a photo-thermoelectric current, because the Seebeck coefficient varies across the interface. In this way, we interpret the sign change of the ultrafast dip when the laser is moved from one electrode to the other [cf. Fig. S9c]. We note that the laser spot at the investigated mid-infrared wavelength is ca. 50 µm.

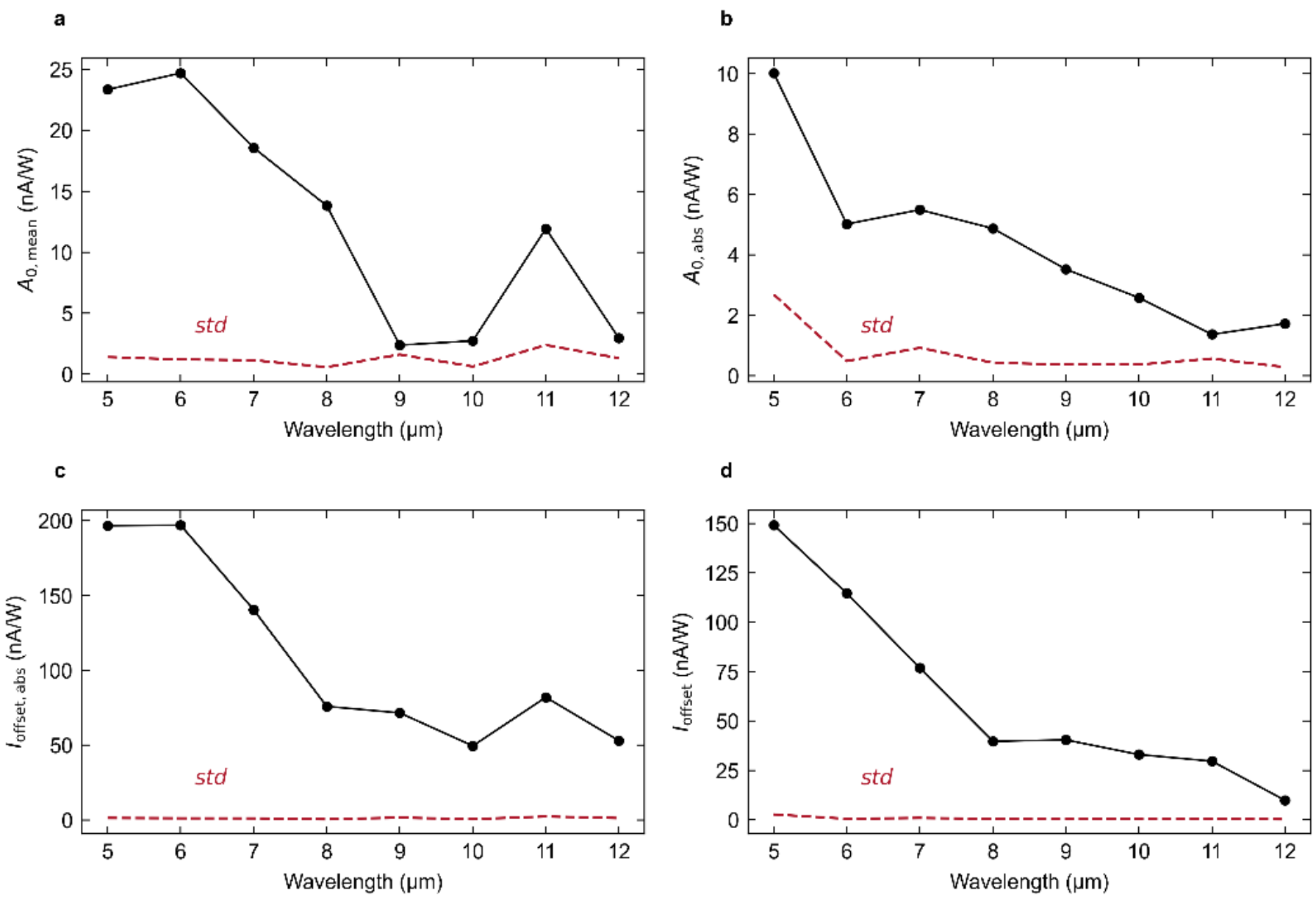

Supporting Figure 10. Mean value of the fitted amplitude A_0 of the time-resolved photocurrent and the standard deviation of the noise for all gate voltages investigated vs. photon wavelength (cf. Figure 2*a* of the main manuscript) for sample “Graphene 1” (a) and sample “Graphene 2” (b) as listed in the Supporting Table 1. (c) and (d): Corresponding mean value of the fitted I_{offset} (cf. Figure 2c) and the standard deviation of the noise vs. photon wavelength.

c)

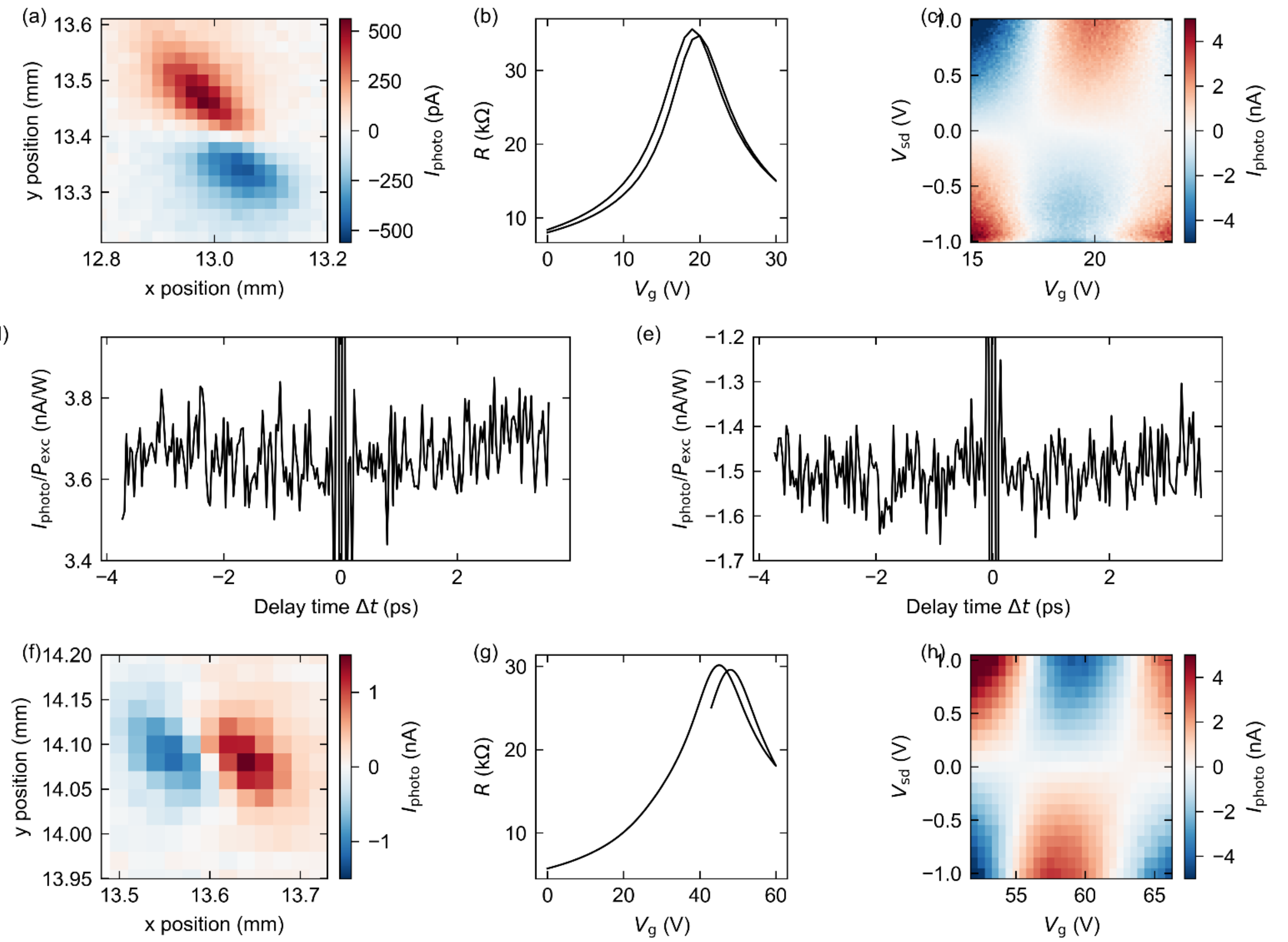

Supporting Figure 11. Single-gate samples in the regime of a bolometric photoresponse. (a) Spatially resolved time-integrated photocurrent map for sample Graphene 4 at V_{sd} = 0 V. (b) Transfer curve for sample Graphene 4. (c) Gate-Bias photocurrent map for sample Graphene 4. (d) Ultrafast photocurrent measurement for charge neutrality point CNP – 10 V. (e) Ultrafast photocurrent measurement for charge neutrality point CNP + 10 V. (f)-(h) Reproduced (a)-(c) for sample Graphene 6.

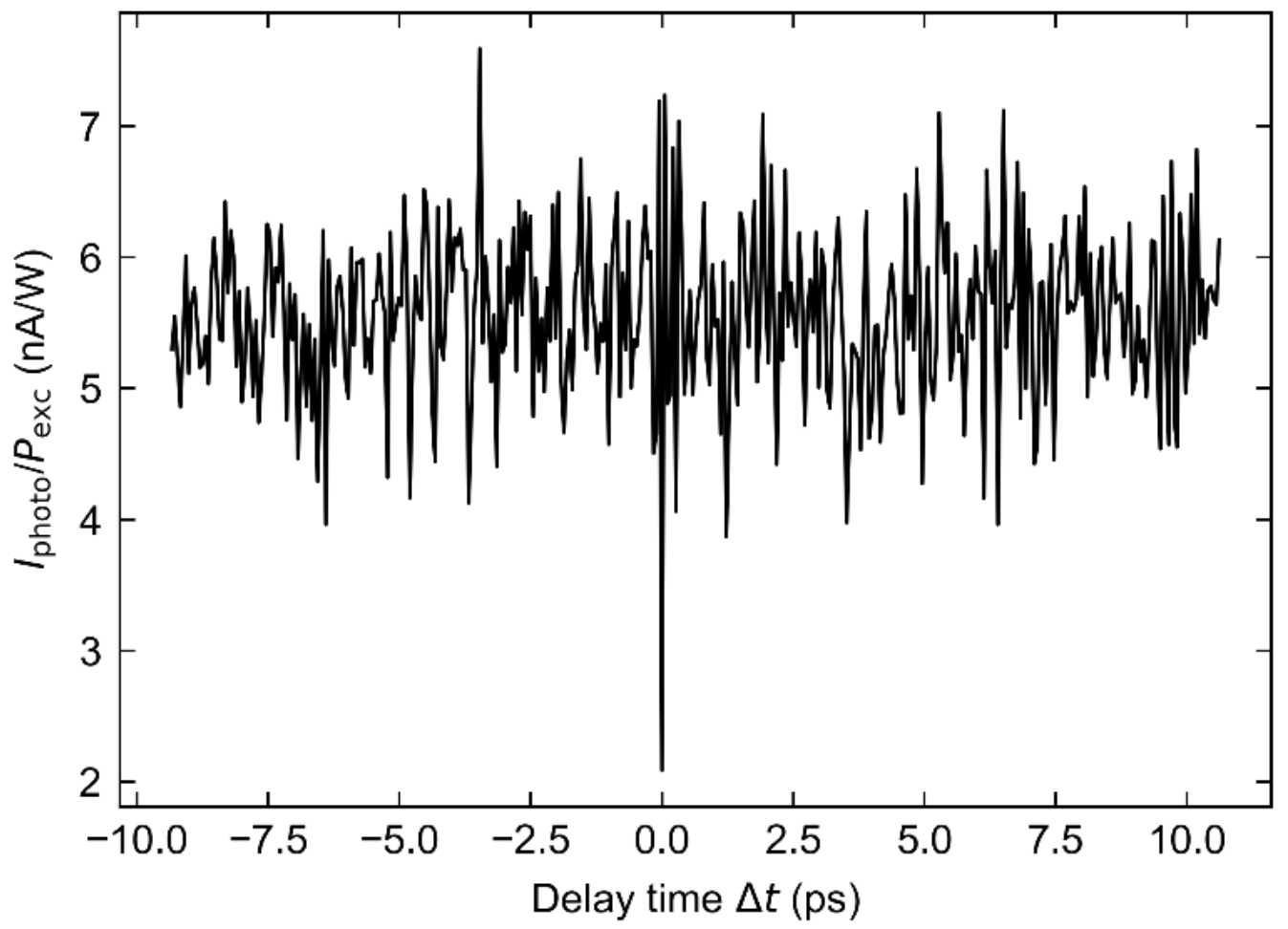

Supporting Figure 12. Ultrafast photocurrent measurement for sample Graphene 7 (CVD on SiC).

Supporting Note 1. Experimental details.

Device fabrication. The devices were fabricated on Si wafers with a 285 nm thermally grown SiO_2 layer. Split gates were patterned on the substrate using optical lithography and metal evaporation of 3 nm platinum (Pt) followed by lift-off in acetone. A ~60 nm thick flake of hexagonal boron nitride (hBN) was mechanically exfoliated from bulk crystals and transferred onto the split gates using a dry transfer technique. Monolayer graphene, obtained by mechanical exfoliation from natural graphite, was identified by optical contrast and Raman spectroscopy, and subsequently transferred on top of the hBN. Source and drain contacts were defined by maskless UV-lithography, followed by deposition of 5 nm Ti and 150 nm Au using electron-beam evaporation, and lift-off in acetone. For the comparison sample of epitaxial graphene (EG), the graphene was prepared on silicon carbide (SiC) via thermal decomposition of SiC. Clean SiC substrates were first etched in 10% H_2/Ar mixture at 1400 °C and 700 Torr for 30 min. The substrates were then heated to 1800 °C in pure Ar at 700 Torr for 20 min to form monolayer EG.

Electrical measurements without photoexcitation. Dark electronic transport measurements were performed at room temperature in a nitrogen-purged environment. The gate voltages were applied to the split gates via a source-measure unit (SMU), and the resulting current through the graphene channel was recorded with a multimeter in combination with a transimpedance amplifier. For two-dimensional transfer maps, both split gate voltages were swept simultaneously over the desired range while recording the current.

Photocurrent measurements with only the pump-pulse. Photocurrent measurements were performed by focusing a tunable mid-IR laser beam onto the center of the graphene using an off-axis parabolic mirror. The laser beam had a diameter of about 50 µm at the plane of the sample for all wavelengths, and it was modulated at 537 Hz using an optical chopper. The laser is the same as the pump-beam for the pump-probe measurements. The resulting photocurrent was detected with a transimpedance amplifier coupled to a lock-in amplifier, referenced to the chopper frequency.

Ultrafast pump–probe measurements. For time-resolved photocurrent measurements, the mid-IR beam was split into a pump and a mechanically delayed probe arm using a beamsplitter. The beams were recombined and focused collinearly onto the graphene channel. The photocurrent as a function of pump–probe delay was recorded with the same lock-in detection scheme as described above.

Transfer Matrix Method (TMM) model. We model the propagation of light through stratified, planar multilayer structures using the Transfer Matrix Method (TMM). At each interface between layers, continuity of the tangential components of the electric and magnetic fields is enforced via Fresnel boundary conditions. Each individual layer of refractive index $n'_j = n_j + i\kappa_j$[4–7] and thickness d_j is represented by a characteristic matrix that relates field amplitudes on either side. The graphene layer is modeled by a surface conductivity. The overall response of the structure is obtained by multiplying the matrices to form the total system matrix. From this system matrix, the reflection and transmission coefficients are calculated[8,9]. We note that the graphene layer is treated to have a conductivity $\sigma = e^2/(4\hbar)$ within the transfer matrix formalism. Most importantly, the model calculation allows us to explain the decrease of photocurrent amplitude in the wavelength range of 5 to 9 µm, since the reflection of the overall heterostack increases.

Supporting Note 2. Model Hamiltonian and charge transport approach.

We model the graphene system by a tight-binding model including the electron-phonon coupling (EPC) based on the Holstein-Peierls Hamiltonian

$$\hat{H} = \sum_{\langle M,N\rangle}\gamma\hat{c}_M^\dagger\hat{c}_N + \sum_{\langle M,N\rangle\mathbf{Q}}\hbar\omega_{\mathbf{Q}}g_{MN}^{\mathbf{Q}}\left(\hat{b}_{\mathbf{Q}}^\dagger + \hat{b}_{-\mathbf{Q}}\right)\hat{c}_M^\dagger\hat{c}_N + \sum_{\mathbf{Q}}\hbar\omega_{\mathbf{Q}}\left(\hat{b}_{\mathbf{Q}}^\dagger\hat{b}_{\mathbf{Q}} + \frac{1}{2}\right), \quad \text{(S1)}$$

where γ is the nearest-neighbor transfer integral of 2.7 eV, $\hbar\omega_{\mathbf{Q}}$ are the phonon energies with the combined index $\boldsymbol{Q} = (\mathbf{q},\lambda)$ with the phonon wave vector $\mathbf{q}$ and mode index λ. The electron-phonon coupling (EPC) is described by the interatomic coupling constants $g_{MN}^{\mathbf{Q}}$. Using the AB-sublattice coordinates of the primitive unit cell of the honeycomb lattice, the Hamiltonian can be rewritten as

$$\hat{H} = \sum_{\langle i,j\rangle}\gamma(\hat{c}_{iA}^\dagger\hat{c}_{jB} + H.c.) + \sum_{\langle i,j\rangle\mathbf{Q}}\hbar\omega_{\mathbf{Q}}\left(\hat{b}_{\mathbf{Q}}^\dagger + \hat{b}_{-\mathbf{Q}}\right)(g_{iAjB}^{\mathbf{Q}}\hat{c}_{iA}^\dagger\hat{c}_{jB} + H.c.) + \sum_{\mathbf{Q}}\hbar\omega_{\mathbf{Q}}\left(\hat{b}_{\mathbf{Q}}^\dagger\hat{b}_{\mathbf{Q}} + \frac{1}{2}\right), \quad \text{(S2)}$$

which is most convenient for the discussion of the model for the EPC below.

Instead of using the full microscopic expressions with a large number of parameters, we introduce a compact model form for the wave-vector dependence of the coupling constants $g_{iAjB}^{\mathbf{Q}}$:

$$g_{iAjB}^{\mathbf{Q}} = \frac{g_{iAjB}^{\mathbf{q}=0,\lambda}}{2\sqrt{N_\Omega}}\left(\alpha_\lambda e^{i\mathbf{q}\cdot\mathbf{R}_i} + \beta_\lambda e^{i\mathbf{q}\cdot\mathbf{R}_j}\right), \tag{S3}$$

The coefficients $g_{iAjB}^{\mathbf{q}=0,\lambda}$, α_λ, and β_λ describe the particular form of the mode-dependent coupling, which will be related to previous models of the EPC in graphene in literature below.[10,11] We further exploit their translational invariance, i.e., using $g_{iAjB}^{\mathbf{Q}} = g_{0A(j-i)B}^{\mathbf{Q}} = g_{0AlB}^{\mathbf{Q}}$ and express the Hamiltonian in the electronic momentum coordinates, using the transformation

$$\hat{c}_{iA} = \frac{1}{\sqrt{N_\Omega}}\sum_{\mathbf{k}} e^{-i\mathbf{k}\cdot\mathbf{R}_i}\hat{c}_{\mathbf{k}A}, \tag{S4}$$

$$\hat{c}_{jB} = \frac{1}{\sqrt{N_\Omega}}\sum_{\mathbf{k}} e^{-i\mathbf{k}\cdot\mathbf{R}_j}\hat{c}_{\mathbf{k}B}, \tag{S5}$$

which yields

$$\hat{H} = \sum_{\mathbf{k}l}\gamma\left(\hat{c}_{\mathbf{k}A}^\dagger\hat{c}_{\mathbf{k}B}e^{i\mathbf{k}\cdot\mathbf{R}_{0l}} + H.c.\right) + \sum_{\mathbf{Q}\mathbf{k}l}\hbar\omega_{\mathbf{Q}}\left(\hat{b}_{\mathbf{Q}}^\dagger + \hat{b}_{-\mathbf{Q}}\right)\left(\hat{c}_{\mathbf{k}A}^\dagger\hat{c}_{\mathbf{k}+\mathbf{q}B}\frac{g_{iAjB}^{\mathbf{q}=0,\lambda}}{2\sqrt{N_\Omega}}\left(\alpha_\lambda e^{i(\mathbf{k}+\mathbf{q})\cdot\mathbf{R}_{0l}} + \beta_\lambda e^{i\mathbf{k}\cdot\mathbf{R}_{0l}}\right) + H.c.\right) + \sum_{\mathbf{Q}}\hbar\omega_{\mathbf{Q}}\left(\hat{b}_{\mathbf{Q}}^\dagger\hat{b}_{\mathbf{Q}} + \frac{1}{2}\right). \tag{S6}$$

The phonon modes of interest here are the two optical Γ-point modes of graphene with mode energies at around 180 meV to 200 meV depending on the literature. These modes are referred to as longitudinal optical (LO) and transverse optical (TO) modes, and possess mode patterns either aligned with the zig-zag direction (LO) or the armchair direction (TO) at the Γ-point of the phonon Brillouin zone (see Supporting Figure 13).[12,13]

We evaluate the Hamiltonian at $\mathbf{q} = 0$, which is used in the calculations for the relaxation time in the following form:

$$\hat{H} = \sum_{\mathbf{k}}\gamma\left(\hat{c}_{\mathbf{k}A}^\dagger\hat{c}_{\mathbf{k}B}\left(1 + e^{i\mathbf{k}\cdot\mathbf{a}_1} + e^{i\mathbf{k}\cdot\mathbf{a}_2}\right) + H.c.\right) + \sum_{\mathbf{k}}\hbar\omega_{\mathrm{LO}}g_{\mathrm{LO}}\left(\hat{b}_{\mathbf{q}=0}^\dagger + \hat{b}_{-\mathbf{q}=0}\right)\left(\hat{c}_{\mathbf{k}A}^\dagger\hat{c}_{\mathbf{k}B}\left(e^{i\mathbf{k}\cdot\mathbf{a}_1} - e^{i\mathbf{k}\cdot\mathbf{a}_2}\right) + H.c.\right) + \hbar\omega_{\mathrm{LO}}\left(\hat{b}_{\mathbf{q}=0}^\dagger\hat{b}_{\mathbf{q}=0} + \frac{1}{2}\right), \tag{S7}$$

for the LO mode and

$$\hat{H} = \sum_{\mathbf{k}}\gamma\left(\hat{c}_{\mathbf{k}A}^\dagger\hat{c}_{\mathbf{k}B}\left(1 + e^{i\mathbf{k}\cdot\mathbf{a}_1} + e^{i\mathbf{k}\cdot\mathbf{a}_2}\right) + H.c.\right) + \sum_{\mathbf{k}}\hbar\omega_{\mathrm{TO}}g_{\mathrm{TO}}\left(\hat{b}_{\mathbf{q}=0}^\dagger + \hat{b}_{-\mathbf{q}=0}\right)\left(\hat{c}_{\mathbf{k}A}^\dagger\hat{c}_{\mathbf{k}B}\left(1 - \frac{e^{i\mathbf{k}\cdot\mathbf{a}_1}}{2} - \frac{e^{i\mathbf{k}\cdot\mathbf{a}_2}}{2}\right) + H.c.\right) + \hbar\omega_{\mathrm{TO}}\left(\hat{b}_{\mathbf{q}=0}^\dagger\hat{b}_{\mathbf{q}=0} + \frac{1}{2}\right) \tag{S8}$$,

for the TO mode, where $\mathbf{a}_1$ and $\mathbf{a}_2$ are the lattice vectors. The effective modes are chosen to have mode energies $\hbar\omega_{\mathrm{LO}} = \hbar\omega_{\mathrm{TO}} =$ 189 meV and moderate dimensionless coupling constant of $g_{\mathrm{LO}} = g_{\mathrm{TO}} =$0.159 yielding an interaction strength of $\hbar\omega_{\mathrm{LO}}g_{\mathrm{LO}} = \hbar\omega_{\mathrm{TO}}g_{\mathrm{TO}} =$30 meV. These couplings at the Dirac point result in reorganization energies of $\Lambda_{\mathrm{LO}} = 3\hbar\omega_{\mathrm{LO}}g_{\mathrm{LO}}^2 =$ 14.3 meV and $\Lambda_{\mathrm{TO}} = \frac{9}{4}\hbar\omega_{\mathrm{TO}}g_{\mathrm{TO}}^2 =$ 10.7 meV, respectively.

The Hamiltonians in Eqs. (S7) and (S8) have been implemented in a highly efficient charge-transport code[14–16], which we use to calculate the carrier relaxation time, that in turn is based on the dc-conductivity

$$\sigma_{\alpha\alpha}^{\mathrm{dc}}(T) = \frac{e^2}{V}\frac{\beta}{2}\lim_{t\to\infty}\frac{d}{dt}\Delta x_\alpha^2(t), \tag{S9}$$

with the thermally averaged mean-square displacement (MSD)

$$\Delta x_\alpha^2(t) = \mathrm{Tr}\left(\hat{\rho}\Delta\hat{x}_\alpha^2(t)\right), \tag{S10}$$

where $\hat{\rho}$ is the grand-canonical density operator $\hat{\rho} = \frac{e^{-\beta(\hat{H}-\mu\hat{N})}}{Z_{\mathrm{gc}}}$.

At zero temperature and in the non-interacting case, the dc-conductivity is then given by[17]

$$\sigma_{\alpha\alpha}^{\mathrm{dc},0} = \lim_{T\to 0}\sigma_{\alpha\alpha}^{\mathrm{dc},0}(T) = \frac{e^2 D(E)v_{\mathrm{F}}^2}{2}\tau_0(E), \tag{S11}$$

where $D(E)$ is the density of states of graphene and v_{F} is the Fermi velocity $v_{\mathrm{F}}=\frac{3}{2}\gamma a_0$, with the C-C distance of $a_0 = 1.42$ Å. To obtain the relaxation time of the interacting system, we thus may define it similarly to the non-interacting case via:

$$\lim_{T\to 0}\sigma_{\alpha\alpha}^{\mathrm{dc}}(T) = \frac{e^2 D(E_{\mathrm{F}}) v_{\mathrm{F}}^2}{2}\tau(E), \tag{S12}$$

which allows to determine the relaxation time from the evaluation of $\sigma_{\alpha\alpha}^{\mathrm{dc}}(T)$. The dc-conductivity is calculated numerically for both the interacting and the non-interacting case. Dividing the dc-conductivities by the corresponding DOS $D(E)$, we obtain the relaxation time as:

$$\tau(E) = 2\frac{\lim_{T\to 0}\sigma_{\alpha\alpha}^{\mathrm{dc}}(T)}{e^2 D(E) v_{\mathrm{F}}^2} = \frac{\lim_{t\to\infty}\frac{d}{dt}\Delta x^2(E,t)}{V D(E) v_{\mathrm{F}}^2}, \tag{S13}$$

which can be compared for both cases. In Supporting Note 3, we discuss the results of our charge transport calculations based on the present model Hamiltonian, including the EPC to the optical modes of graphene.

Supporting Note 3. Polaron dressing of ballistic electrons at ultrafast time scales

After introducing the model Hamiltonian and transport approach to determining carrier relaxation times, we compare our theoretical results to the experimental data. In the charge-transport simulations, we calculate the expectation value of the MSD of the hot electrons (assuming no thermal phonon occupation) in the zero-phonon subspace $|0\rangle_{\mathrm{ph}}$

$$\Delta x_\alpha^2(t) = \mathrm{Tr}_{\mathrm{el}}\big(\langle 0|_{\mathrm{ph}}\hat{\rho}\Delta\hat{x}_\alpha^2(t)|0\rangle_{\mathrm{ph}}\big). \tag{S14}$$

The results are shown in Figure 4 of the main text. Fig. 4b displays the carrier relaxation times defined in Eq. (S13) as function of energy E, taken as half the experimental excitation energy (cf. Fig. 4a). Without EPC (black curve), the electrons propagate ballistically $\Delta x_\alpha^2(t) \propto t^2$, since no other scattering mechanisms or disorders are considered in this case, up to the total simulation time set to τ_{ref}= 3.7 ps here and for all subsequent simulations. This simulation time effectively acts as coherence-limiting time (decoherence time) for all subsequent simulations, where the coherent quantum dynamics would stop by inelastic scattering mechanisms that set in, such as supercollisions. For the disorder- and phonon-free limit, the MSD follows approximately $\Delta x_\alpha^2(t) \approx \frac{e^2 D(E) v_{\mathrm{F}}^2}{2}\tau_{\mathrm{ref}}^2$ yielding $\tau(E) \approx \tau_{\mathrm{ref}}$ shown as analytical reference in Fig. 4b (grey dashed line). The numerical results (black curve) agree with this analytic behavior, aside from small finite-size fluctuations due to the finite system size of $1.6\mathrm{x}10^7$ electronic orbitals.

Including LO and TO optical phonons changes the picture markedly. The relaxation time (S13) (blue curve) drops sharply from 3.7 ps to about 1.6 ps near E = 0.1 eV, followed by a gradual and eventually full recovery of the reference result at around E = 0.4 eV. We observe that the dynamics are ballistic at this time scale ($\Delta x_\alpha^2(t) \propto \tau_{\mathrm{ref}}^2$), indicating that the dip is not caused by phonon-scattering but by coherent polaron dressing (Fig. 4a). This polaronic dressing renormalizes the band velocity (analogous to a mass enhancement) with a maximum renormalization factor of ~0.5 (given by τ/τ_{ref}). Hence the dip of the carrier relaxation time in Fig. 4b is thus a signature of the coherent, ballistic propagation of the dressed electron before scattering sets in. Indeed, the polaron-dressing occurs at much earlier times, specifically at the oscillation period of the optical phonons (≈20 fs). The suppression of dressing at higher energies E is attributed to faster pseudospin (sublattice) precession ($\propto |\mathbf{k}|$). The velocity renormalization becomes less effective and at higher energies (~0.22 eV) and even scattering by the optical mode is so inefficient that the EPC-free ballistic limit of the MSD is fully recovered.

Improvements to the effective model of optical $\mathbf{q} = 0$ modes towards more refined sets of microscopic EPC parameters are conceivable, but the present EPC Hamiltonian model and the considered transport scenario clearly reproduce the experimental findings for the energy dependence of the carrier relaxation time. As a result, we can attribute the drop in the experimental relaxation time to energy-dependent polaron dressing due to the EPC of the optical modes.

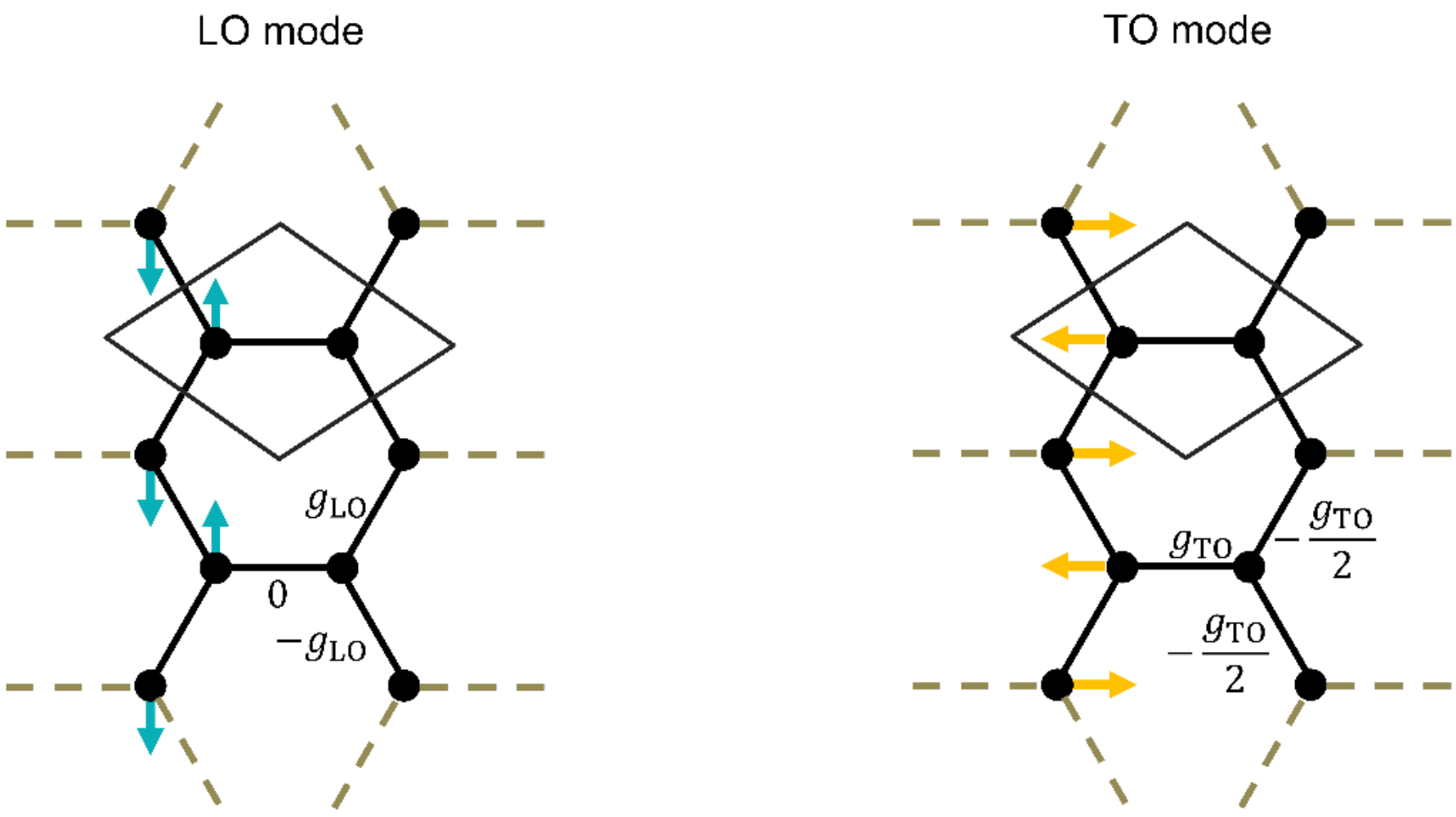

Supporting Figure 13. Mode patterns of the Γ-point optical phonons for LO and TO modes in the real space. The dimensionless EPC constants g_{LO} and g_{TO} (see Eqs. (S7) and (S8)) follow this coupling pattern for the nearest neighbors in real space.

References:

[1] J.C.W. Song, M.S. Rudner, C.M. Marcus, and L.S. Levitov, "Hot Carrier Transport and Photocurrent Response in Graphene," Nano Lett. **11**(11), 4688–4692 (2011).

[2] A. Urich, K. Unterrainer, and T. Mueller, "Intrinsic Response Time of Graphene Photodetectors," Nano Lett. **11**(7), 2804–2808 (2011).

[3] D. Sun, G. Aivazian, A.M. Jones, J.S. Ross, W. Yao, D. Cobden, and X. Xu, "Ultrafast hot-carrier-dominated photocurrent in graphene," Nature Nanotech **7**(2), 114–118 (2012).

[4] D.V. Grudinin, G.A. Ermolaev, D.G. Baranov, A.N. Toksumakov, K.V. Voronin, A.S. Slavich, A.A. Vyshnevyy, A.B. Mazitov, I.A. Kruglov, D.A. Ghazaryan, A.V. Arsenin, K.S. Novoselov, and V.S. Volkov, "Hexagonal boron nitride nanophotonics: a record-breaking material for the ultraviolet and visible spectral ranges," Mater. Horiz. **10**(7), 2427–2435 (2023).

[5] A.D. Rakić, A.B. Djurišić, J.M. Elazar, and M.L. Majewski, "Optical properties of metallic films for vertical-cavity optoelectronic devices," Appl. Opt., AO **37**(22), 5271–5283 (1998).

[6] D. Franta, D. Nečas, I. Ohlídal, and A. Giglia, "Optical characterization of SiO2 thin films using universal dispersion model over wide spectral range," in *Optical Micro- and Nanometrology VI*, (SPIE, 2016), pp. 253–267.

[7] D. Franta, A. Dubroka, C. Wang, A. Giglia, J. Vohánka, P. Franta, and I. Ohlídal, "Temperature-dependent dispersion model of float zone crystalline silicon," Applied Surface Science **421**, 405–419 (2017).

[8] L. Sigl, M. Troue, M. Katzer, M. Selig, F. Sigger, J. Kiemle, M. Brotons-Gisbert, K. Watanabe, T. Taniguchi, B.D. Gerardot, A. Knorr, U. Wurstbauer, and A.W. Holleitner, "Optical dipole orientation of interlayer excitons in MoSe2 − WSe2 heterostacks," Phys. Rev. B **105**(3), 035417 (2022).

[9] H. Benisty, R. Stanley, and M. Mayer, "Method of source terms for dipole emission modification in modes of arbitrary planar structures," J. Opt. Soc. Am. A, JOSAA **15**(5), 1192–1201 (1998).

[10] A.H. Castro Neto, and F. Guinea, "Electron-phonon coupling and Raman spectroscopy in graphene," Phys. Rev. B **75**(4), 045404 (2007).

[11] M. Sabbaghi, T. Stauber, H.-W. Lee, J.S. Gomez-Diaz, and G.W. Hanson, "In-plane optical phonon modes of current-carrying graphene," Phys. Rev. B **105**(23), 235405 (2022).

[12] K.M. Borysenko, J.T. Mullen, E.A. Barry, S. Paul, Y.G. Semenov, J.M. Zavada, M.B. Nardelli, and K.W. Kim, "First-principles analysis of electron-phonon interactions in graphene," Phys. Rev. B **81**(12), 121412 (2010).

[13] A. Jorio, L.G. Cançado, and L.M. Malard, "Vibrations in Graphene," in *2D Materials: Properties and Devices*, edited by P. Avouris, T. Low, and T.F. Heinz, (Cambridge University Press, Cambridge, 2017), pp. 71–89.

[14] Z. Fan, J.H. Garcia, A.W. Cummings, J.E. Barrios-Vargas, M. Panhans, A. Harju, F. Ortmann, and S. Roche, "Linear scaling quantum transport methodologies," Physics Reports **903**, 1–69 (2021).

[15] M. Panhans, and F. Ortmann, "Efficient Time-Domain Approach for Linear Response Functions," Phys. Rev. Lett. **127**(1), 16601 (2021).

[16] M. Panhans, S. Hutsch, and F. Ortmann, "Insight on charge-transfer regimes in electron-phonon coupled molecular systems via numerically exact simulations," Commun Phys **6**(1), 125 (2023).

[17] E.H. Hwang, and S. Das Sarma, "Acoustic phonon scattering limited carrier mobility in two-dimensional extrinsic graphene," Phys. Rev. B **77**(11), 115449 (2008).
